# Next-generation engineered nanogold for multimodal cancer therapy and imaging: a clinical perspectives

**DOI:** 10.1186/s12951-022-01402-z

**Published:** 2022-07-02

**Authors:** Madhusudhan Alle, Garima Sharma, Seung-Hwan Lee, Jin-Chul Kim

**Affiliations:** 1 Institute of Forest Science, Kangwon National University, Chuncheon, 24341 Republic of Korea; 2 Department of Biomedical Science & Institute of Bioscience and Biotechnology, Kangwon National University, Chuncheon, 24341 Republic of Korea; 3 Department of Forest Biomaterials Engineering, College of Forest and Environmental Sciences, Kangwon National University, Chuncheon, 24341 Republic of Korea

**Keywords:** Nanogold, Cancer therapy, Multimodal approaches, Theranostic, Clinical studies

## Abstract

Cancer is one of the significant threats to human life. Although various latest technologies are currently available to treat cancer, it still accounts for millions of death each year worldwide. Thus, creating a need for more developed and novel technologies to combat this deadly condition. Nanoparticles-based cancer therapeutics have offered a promising approach to treat cancer effectively while minimizing adverse events. Among various nanoparticles, nanogold (AuNPs) are biocompatible and have proved their efficiency in treating cancer because they can reach tumors via enhanced permeability and retention effect. The size and shape of the AuNPs are responsible for their diverse therapeutic behavior. Thus, to modulate their therapeutic values, the AuNPs can be synthesized in various shapes, such as spheres, cages, flowers, shells, prisms, rods, clusters, etc. Also, attaching AuNPs with single or multiple targeting agents can facilitate the active targeting of AuNPs to the tumor tissue. The AuNPs have been much explored for photothermal therapy (PTT) to treat cancer. In addition to PTT, AuNPs-based nanoplatforms have been investigated for combinational multimodal therapies in the last few years, including photodynamic therapy, chemotherapy, radiotherapy, immunotherapy, etc., to ablate cancer cells. Thus, the present review focuses on the recent advancements in the functionalization of AuNPs-based nanoconstructs for cancer imaging and therapy using combinatorial multimodal approaches to treat various cancers.

**Graphical Abstract:**

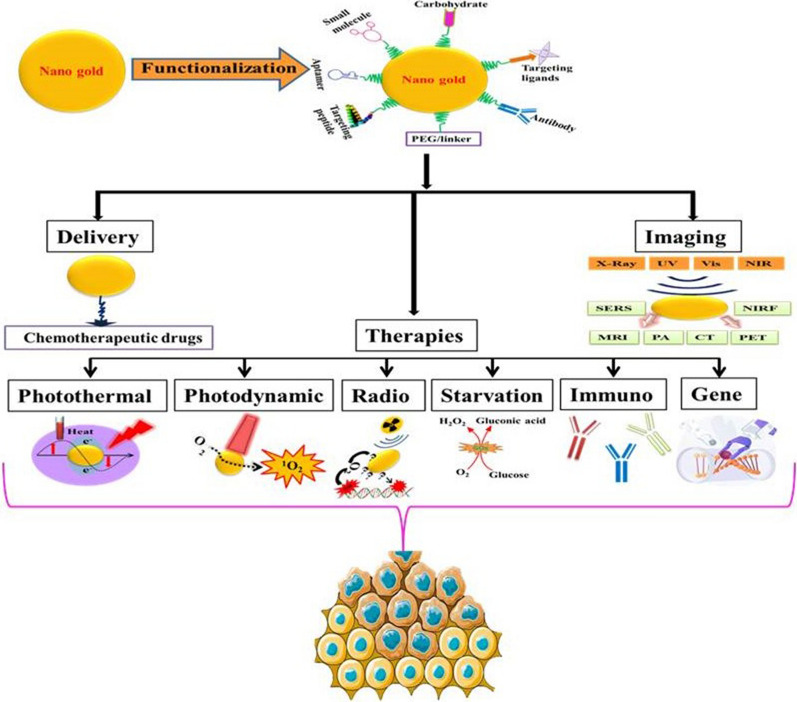

## Introduction

The worldwide statistics of cancer-associated mortalities and morbidities continue to increase despite the advances in surgery, chemotherapy (CTX), immunotherapy, and radiotherapy (RT) [1, 2]. The increase in cancer-related deaths is possibly due to the therapy-associated side effects owing to the lack of specificity and selectivity. In addition, the complexity and recurrence of the tumor are also possible reasons for the high mortality rate in cancer patients [3]. Nowadays, hyperthermia-mediated cancer therapies, such as microwave radiation, ultrasound, photothermal therapy (PTT), and photodynamic therapy (PDT), are gaining significant interest. However, similar to other conventional cancer therapies, these cancer therapies are also not tumor-targeted, and thus they may cause adverse effects. Thus, there is a need to develop novel therapeutic strategies with reduced side effects and enhanced clinical significance.

Nanomedicines have made significant contributions to cancer therapy, prevention, and diagnosis in the last few decades. Various new nanomaterials, such as polymers, liposomes, quantum dots, dendrimers, and inorganic nanoparticles, have been explored for cancer diagnosis and treatment [4, 5]. The multifunctional nanosystems that combine diagnosis and treatment are known as theranostics nanoparticles. Thus, nanobiotechnology has shown a multitude of potential in cancer theranostics that might meet the medical challenges offered by conventional cancer treatments [6]. Among these nanomaterials, inorganic nanoparticles are extensively explored for cancer diagnosis and treatment [7–9]. Since conceptualizing nanomaterials for cancer diagnosis and therapy, the studies on nanogold (AuNPs)-based cancer treatment have extensively progressed. This is possible because AuNPs are easy to synthesize, cost-effective, have a large surface-to-volume ratio, penetrate the biological tissues, and have inherent biocompatibility [10]. In addition, the AuNPs are less toxic and can enter tumor cells via enhanced permeability and retention (EPR) effect [11].

In the last few decades, the AuNPs were surface-functionalized by various molecules, such as peptides, folate, ligands, and antibodies, for targeted delivery to the local tumor site [12]. In addition, AuNPs were attached to chemotherapeutic drugs and drug-loaded stimuli-sensitive polymeric nanoparticles for stimuli-responsive drug release [13]. Thus, targeted delivery of AuNPs to the tumor site, stimuli-responsive drug release, biocompatibility, improved stability, and solubility of drugs loaded on AuNPs are suitable candidates for cancer theranostics with reduced morbidity and mortality risks. Thus, suggesting combining multiple therapies at a single platform to achieve significant antitumor responses compared to monotherapies. For instance, inducing hyperthermia in cancer could increase the sensitivity of cancer cells towards chemotherapeutic drugs and radiations, indicating a synergistic therapeutic approach [14]. Combining imaging or diagnostic agents with multiple therapies also collaborates with win/win outcomes.

**Scheme 1:**
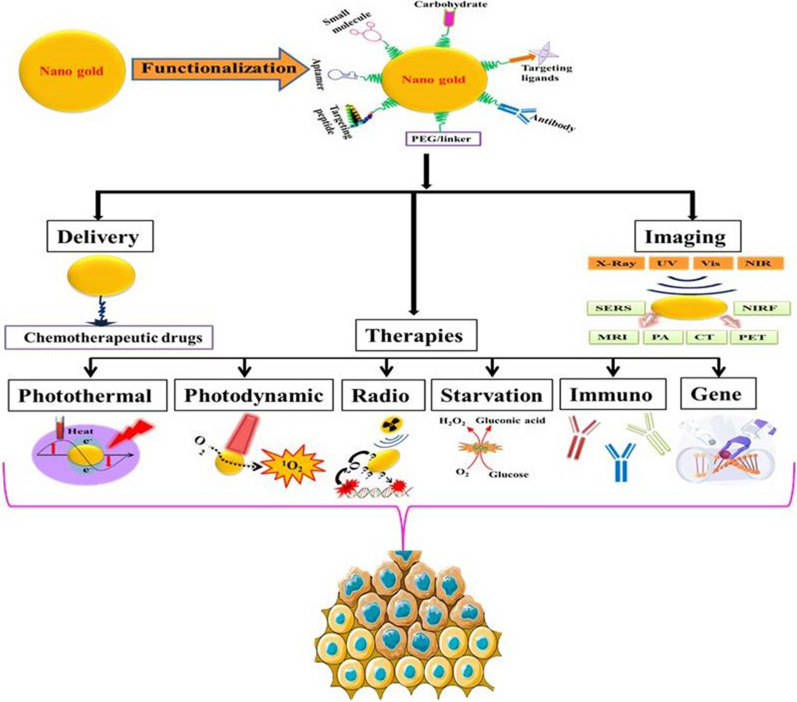
Illustrates the use of functionalized AuNPs for combinational multimodal theranostics

The PubMed alone covers more than 1000 articles in less than five years (2018-early 2022) on this subject (Keywords: gold nanoparticles, cancer treatment, functionalization). These studies are of tremendous importance as they demonstrate remarkable efforts on AuNPs-based multimodal cancer theranostics. Since these articles are important to discuss the recent advancements, the present review systematically summarizes the representative findings on the functionalization and designing of AuNPs for multimodal cancer theranostics published in the last 4–5 years (Scheme 1). This review also includes a summary of the factors affecting the therapeutic role of AuNPs and the mechanistic role of AuNPs in various types of therapies. Further, we also discuss the clinical studies performed for AuNPs-based cancer ablation therapies.

### Factors affecting AuNPs based cancer therapy

It has been widely investigated that the biological and optical properties of AuNPs are strongly dependent on the size, shape, and charge of AuNPs-based nanoconstructs [15]. Thus, the biological properties of AuNPs can be regulated by tuning the size and shape of AuNPs. The accumulation of AuNPs at the tumor site and cell internalization depends mainly on their size. Large-sized AuNPs (i.e., > 200 nm) have poor tumor tissue penetration and poor cell interaction abilities. Moreover, they are removed from the body system by the liver and spleen. On the contrary, small-sized AuNPs (i.e., < 10 nm) have enhanced tumor tissue penetration but are rapidly cleared from the system by the kidneys and may cause hemolysis [16]. Thus, the optimum AuNPs size for increased circulation time and maximum tumor cell internalization is between 10 and 100 nm (optimum is around 20 nm) [14, 17].

In addition to the size, the surface charge is also a determinant factor for the cellular internalization of AuNPs. It has been observed that positively charged AuNPs are internalized more (around 5–10 times higher) by the cells than neutral or negatively charged AuNPs [18]. This is plausible because the negative cellular membrane might have a high affinity for positively charged AuNPs, resulting in higher adhesion and cellular uptake by generating transient holes in the membrane [19].

AuNPs are the plasmonic nanoparticles with remarkable optical properties because of their ability to absorb and scatter light. The AuNPs interact with light at a specific wavelength resulting in the oscillation of conductive surface electrons, known as localized surface plasmon resonance (LSPR). The LSPR defines the intensities of light absorption and scattering. Size and shape are the two determining factors that are correlated with the frequency of the absorption band of the AuNPs [20]. Since the optical properties of AuNPs are dependent on both the size and shape, the absorption of AuNPs can be regulated by modulating these two determining factors [21]. It has been well-established that the AuNPs can be tuned to various shapes, such as Au nanospheres, Au nanorods (AuNRs), Au nanostars, Au nanocrystals, Au nanoshells (AuNSs), hollow Au nanoparticles (HAuNP), Au nanocluster (AuNCs), Au nanoprisms (AuNPrs) and Au nanocage. Thus, both the biological and optical properties of AuNPs can be modulated by tuning the size, charge, and shape of the nanoparticles.

### AuNPs for cancer photothermal therapy (PTT)

The PTT is a minimally invasive method with minimum side effects among the cancer therapies. It uses near-infrared (NIR) radiation, particularly the NIR-I, and NIR-II at 750–1200 nm wavelength for cancer therapy at precise locations with high efficiency to ablate cancer cells/tissues. The tumor cells are inefficient in dissipating heat because of their abnormal vascular structures, leading to hyperthermia that causes irreversible cellular damages, such as cell membrane disruption and protein denaturation [22, 23]. Thus, The tumor cells are more sensitive to the PTT effect than the healthy tissues, reducing the risk of cytotoxicity to healthy cells.

Compared to higher energy radiations, such as UV-radiation, the NIR light shows rapid recovery and much deeper penetration in the tissue cells [24]. In general, NIR light can penetrate approximately 1 cm deep in the human body. It has been well-known that light scattering reduces when the wavelength increases, resulting in light penetration to the deep tissues [25]. However, the depth of light penetration (i.e., 1–10 cm) is dependent on various factors, such as size and shape of nanoparticles, type of the tissue, time of NIR exposure, the wavelength of NIR, etc., [26].

Nanoparticles with a simple surface functionalization process, plasmon resonance tunability, high photostability, and high photothermal conversion efficiency are preferred for PTT [27]. AuNPs with strong LSPR are suggested for PTT-based cancer treatment among these nanoparticles [28–30]. Due to their unique optical properties, AuNPs have been known to absorb light with high efficiency at the NIR region, at a 700–1350 nm wavelength, and convert them into heat-producing PTT effect [31]. Upon NIR excitation, the SPR of AuNPs generates hot electrons on the nanoparticle surface. The excited electron transfers the absorbed energy in the form of heat to the metallic lattice, which cools by phonon–phonon interactions. The thermal energy is then transferred to the environment [32], increasing the temperature to about 41–47 °C in the cell compartment where the nanoparticles are located. This might possibly cause irreversible damage to the cells or the cellular DNA [33].

The absorption of NIR light at desired wavelength by AuNPs can be tuned via modulating the shape of AuNPs. The plasmonic PTTs of different shaped AuNPs can vary due to variations in their SPR oscillations and the cross-section area of AuNPs. More interestingly, different shapes of the AuNPs exhibit plasmonic adsorption from NIR radiations from different windows. For example, the Au nanospheres show intense plasmonic absorption in the first NIR window, while AuNRs show plasmonic absorption in the second NIR window. For PTT applications, AuNPs with large extinction cross-section, i.e., addition of absorption (C_abs_) and scattering (C_sca_) cross-section area, and high C_abs_/C_sca_ ratio is preferred [34]. Among other shapes of nanogold, AuNRs and Au nanocages have high extinction cross-section and low threshold power. Thus, they are the most preferred shapes for the photothermal destruction of cancer cells [35].

However, a study reported that sharp-tipped AuNPs have higher efficiency for photothermal conversion than other shapes [36]. Au nanobipyramids (AuNBPs) are another type of AuNPs consisting of two pentagonal pyramids and have smaller plasmon peak widths and narrower size and shape distributions, sharper ends than AuNRs [37, 38]. Another study showed that Au nanostars have a unique symmetrical structure and sharp edges that enable the LSPR peak modulation of Au nanostars in the NIR region [39]. Depciuch et al. showed that the PTT effect of Au nanostars depends on edge widths and lengths of the star arms and the values of photothermal efficiency are higher with the increase of the arm lengths, which is correlated with the reducer concentration [40]. Therefore, the dispersion and absorption properties of AuNPs can be changed by tuning the shapes and sizes of AuNPs [41]. Au nanostars have high photothermal conversion efficiency, small size, and facile synthesis, resulting in significant cancer diagnosis and therapy. However, the Au nanostars have poor stability. The stability of Au nanostars is improved by coating any stabilizing agents on their surface. It was found that among different types of AuNPs (such as nanorods, nanostars, and nanocubes), Au nanoprisms (AuNPrs) possess the highest photothermal conversion efficiency [42, 43], and are easily internalized in the cells compared to AuNRs [44]. In addition, AuNPrs are beneficial for long-term biosafety as they can be removed from the tissue faster than smaller AuNRs [45].

Although different-shaped AuNPs act as a promising PTT agent, their application in clinical practice is limited due to their poor photothermal stability, which can be improved by reducing the size of AuNPs and modifying their surface properties for the targeted location site of tumor [46]. The AuNPs lose their photothermal converting ability upon repetitive NIR radiation. In addition, AuNPs have a poor drug loading capacity, limiting the use of AuNPs as drug carriers [47]. Moreover, most of the NPs cannot reach the tumor site due to the hindrance caused by the dense interstitial structure of the tumor and lack vessels in the tumor [48]. Thus, to achieve the maximum localization of AuNPs to the tumor site, targeting ligands (such as antibodies, single-chain fragments of antibodies, carbohydrates, or simplified peptide sequences, etc.) are introduced on the surface of AuNPs [49]. Interestingly, the shape and size of the AuNPs might also affect the active targeting of AuNPs despite the attachment of the targeting ligand.

### AuNPs for photodynamic cancer therapy (PDT)

The PDT requires a photosensitizer (PSs) molecule which excites and reacts with oxygen upon exposure to light in a determined wavelength, generating oxidant species (radicals, singlet oxygen, triplet species) in target tissues leading to cell death. The PDT-mediated cytotoxicity is due to the oxidation of biomolecules, such as nucleic acids, lipids, proteins, etc., present in cells, resulting in altered cell signaling cascades and gene expressions. However, due to the hydrophobicity of most of the currently used PSs, AuNPs are suggested as carriers of PSs. In addition, to act as a carrier for PSs, AuNPs can also enhance the photosensitizing properties of PSs by acting as a PS by generating ROS in response to irradiation [50].

Some of the most widely used PSs for PDT include Chlorin e6 (Ce6), zinc phthalocyanine (ZnPcs), and alphthalocyanine (AlPcS_4_Cl). The Ce6, a second-generation PS, is among the most widely used PS with low toxicity and high efficacy. The Ce6 delivered using AuNPs showed enhanced apoptotic activity in the cancer cells [51]. Another PS (ZnPcs)/AuNPs conjugate also showed enhanced singlet oxygen (^1^O_2_) generation and remarkable PDT in the cancer cell [52]. The effectiveness of PDT against cancer stem cells (CSCs) is also improved by combining PSs, such as AlPcS_4_Cl, with AuNPs and CSCs-targeting antibodies [53]. Wang et al. showed that AlPcS-conjugated AuNBPs could significantly suppress tumor growth with minimal side effects in tumor xenografts [54]. Although AuNBPs have rarely been explored, they have advantages over AuNSs and AuNRs because they can sensitize O_2_ by transferring the energy to attached PSs [54].

5-Aminolevulinic acid (5-ALA) (a Cathepsin E-sensitive (CTSE) PDT therapy prodrug) is a type of PS, which is designed to be activated selectively by endogenous Cathepsin E (Cath E), a proteolytic enzyme highly expressed within the cancer cells. When combined with 5-ALA, AuNPs showed significant PDT efficacy against cancer cells [55]. Thus, custom designing of PSs together with modifications in the shape and size of AuNPs could be a promising approach for enhanced PDT. In another study, IR820, a photosensitive drug, was loaded with ultra-small spherical AuNPs nanoconstructs synthesized using Gadolinium (Gd) (Gd–AuNPS@IR820). They found that Gd–AuNPS@IR820, of hydrodynamic size 72.4 nm, had excellent tumor targeting ability and enhanced tumor ablation properties in hepatocellular carcinoma HCC-LM3 cells bearing nude mice due to enhanced PTT and PDT combinational therapy [56].

**Fig. 1:**
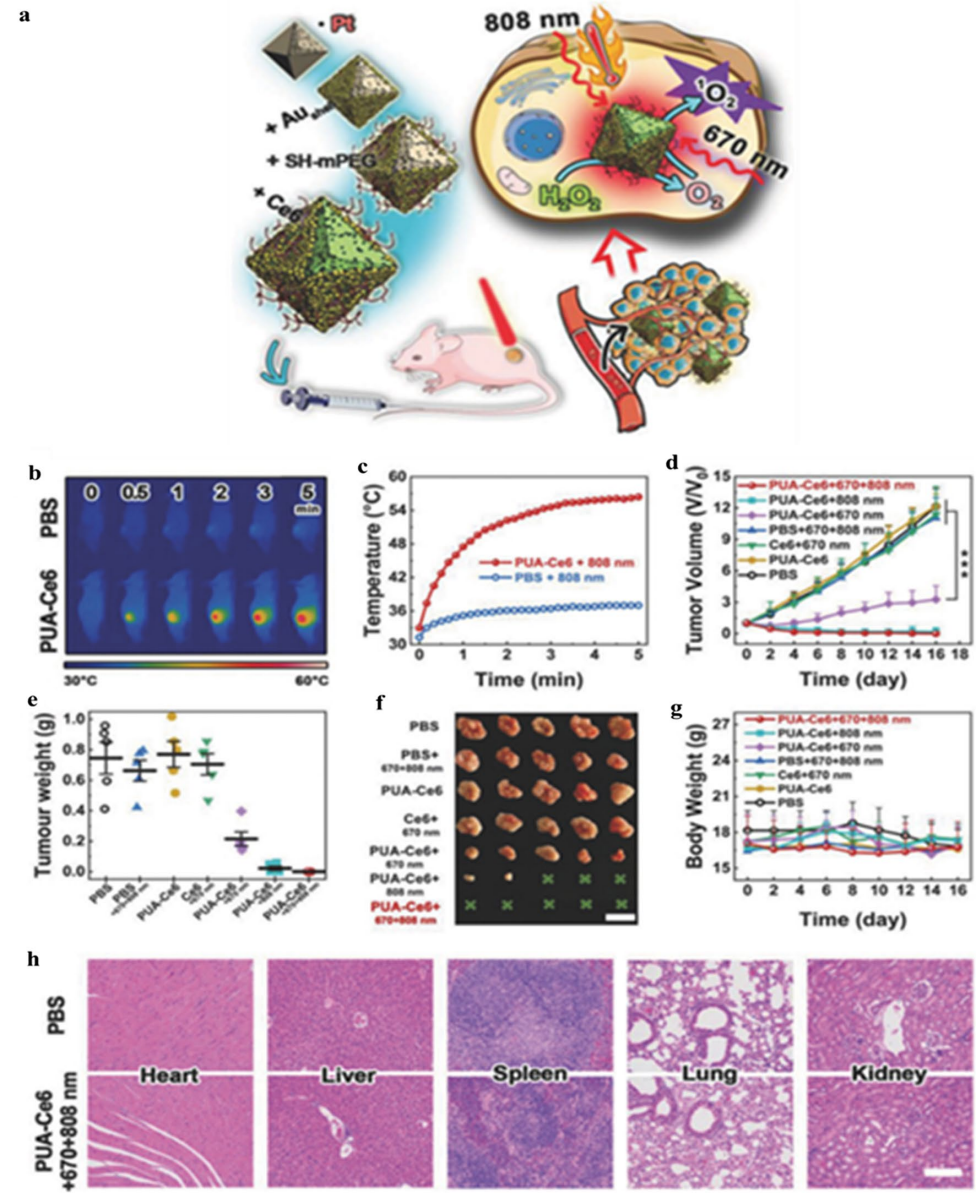
**a** Schematic illustration for the preparation of PUA-Ce6 and its application on a combination of PTT and potential enhanced PDT by converting intratumor H_2_O_2_ into O_2_ for tumor therapy, **b** Infrared thermal images of PBS or PUA-Ce6 nanoparticles-injected MCF-7 tumor-bearing mice under 808 nm laser irradiation (1.0 W/cm^2^), **c** the temperature variation curves of the tumor, **d** tumor volume curves, **e** tumor weight change of mice, **f** tumor photographs, **g** body weight, and **h** H&E-stained images of main organs collected from mice after treatments. Reproduced with permission from [58]. Copyright ©2018, John Wiley

Similarly, AuNRs can be combined with platinum nanoparticles as potential nanophoto‑sensitizers to enhance the PTT effect [57]. Liu et al. used amino-functionalized porous metal–organic frameworks (NH_2_-MOFs) nanoparticles as superior templates for the facile and general one-step method to synthesize porous AuNSs (NH_2_-MOFs@Aushell). Further, they encapsulate platinum nanozymes in NH_2_-MOFs, coated with porous AuNSs coating, and loaded it with Ce6 PS (Pt@UiO-66-NH_2_@Aushell-Ce6) to achieve synergistic PDT and PTT effects (Fig. 1) [58]. Thus, indicating that AuNPs can be combined with other inorganic nanozymes and PSs for an excellent synergistic tumor therapy strategy.

### AuNPs for radiotherapy (RT)

Radiotherapy (RT) is a frequently used method to ablate solid tumors via ionizing radiation-mediated damage to tumor tissue [59]. The ionizing radiations cause cellular damage by generating free radicals via inducing water radiolysis. Although RT is generally used in half of the cancer patients, it is strongly evidenced that RT can cause tumor cell radio-resistance that requires higher radiation doses for cancer treatment [60]. Moreover, high radiation doses may cause damage to normal cells surrounding the tumor tissue [61]. Although RT is known for eradicating local tumor growth by damaging DNA via high-energy ionizing radiation, the therapeutic efficacy of RT is limited due to the problems associated with the delivery of radiation dose to the site of the tumor without harming normal cells. Therefore, increasing damage to tumor tissues while reducing the damage to normal tissues while using RT is desired. Hence, to improve the radiation absorbance, radiosensitizers are suggested, which might increase the RT outcome. Further, it has been suggested that PTT can sensitize RT-resistant cancer cells to enhance anti-tumor efficacy via synergistic effect [62, 63].

Materials with high atomic numbers have high photoelectric absorption cross-sections and emit secondary radiation (i.e., Auger/photoelectrons). Thus, resulting in a high generation of free radicals. Gadolinium, platinum, and iodine are the most widely used radiosensitizers. However, AuNPs offer advantages over these frequently used radiosensitizers, such as high atomic number (i.e., 79), ability to modulate the size for passive accumulation at the tumor site, modulation of shape, and the possibility of attaching active targeting/imaging molecule. Thus, AuNPs can be used as biocompatible radiosensitizers with low toxicity to normal cells [64].

Furthermore, the AuNPs-mediated synergistic PTT and RT therapy can modulate various cellular pathways, activating pro-apoptosis unfolded protein response (UPR) cascades via inhibiting heat shock protein A5 (HSPA5), a member of the HSP70 family, proved to be a promising approach. Upregulation of HSPA5 in cancer cells has been well-documented. HSPA5 aids in the repair of irradiation-induced DNA and protein damage resulting in the development of resistance in tumor cells against various treatments. Moreover, HSPA5 assists in the maintenance of cellular homeostasis via regulating endoplasmic reticulum (ER) stress and activating UPR cascades [65].

Fengrong Zhang et al. developed liposomes-based honeycomb-like AuNPs for combined interventional photothermal and brachytherapy (IPT-BT) (a localized internal RT performed by implanting iodine-125 radioactive seed with minimal invasion). The honeycomb-like AuNPs showed a 96.6% tumor inhibition rate in the SW1990 orthotopic mice model via dsDNA damage, improved O_2_ supply, and better penetration of nanoparticles inside the tumor [66]. Thus, AuNPs can be used for RT to enhance the therapeutic efficacy against cancer.

Another hurdle for using AuNPs for RT is that the standard linear accelerators (LINACs) produce a small proportion of low-energy photons in clinical photon beams [67]. Very recently, Piccolo et al. have overcome this challenge by developing a novel diamond target beam (DTB) that quadruples the proportion of low energy photons and increases the amount of localized Auger electrons from AuNPs [67]. Although this study showed promising enhancement of RT, inadequate AuNPs uptake affected the tumor targeting.

It has been found that the attachment of aptamers [68, 69], peptides [70–73], and antibodies [74–77] endow AuNPs with selective tumor cell internalization capability. Thus, attachment of active targeting agent may further enhance the effectiveness of AuNPs as a radiosensitizer or as a carrier of radiosensitizers against tumor growth.

### AuNPs for immunotherapy and immune cell-based delivery

Studies suggest that hyperthermia-inducing therapeutic strategies can synergize with immunotherapies [78]. For example, unchecking the T-cell activity within the immunosuppressive tumor microenvironment via the immune checkpoint blockade is one of the most promising immunotherapy. In accordance, it was found that combining the plasmonic Au nanostars-mediated PTT with checkpoint blockade immunotherapy improved the therapeutic efficacy in the CTX-2A glioma cell murine model. This system can reject the rechallenge offered by the memorized anti-cancer immune response [79].

In addition, tumor tropic cells, such as platelets, macrophages, mesenchymal stem cells (MSCs), induced pluripotent stem cells (iPSCs), and neural stem cells (NSCs), can be used to enhance the uptake of nanoparticles at the site of tumor [80–84]. Since MSCs could infiltrate and migrate to the entire tumor, Huang et al. loaded MSCs with TAT-conjugated Au nanostars for the enhanced uptake of nanoparticles by the tumor cells [85]. Nowadays, immune cells, such as macrophages [86], T-cells [87], monocytes [88], natural killer (NK) cells, and neutrophils [89, 90], are also exploited for targeting therapeutic agents to the tumor cells. The neutrophils can cross the endothelial barrier and enter the tumor tissue in response to the chemoattractive agents released by the tumor cells. It is known that AuNRs are recognized by tumor-infiltrating innate immune cells and are accumulated at the tumor site, resulting in enhanced tumor ablation upon endoscopic-guided laser irradiation [91]. Although immune cells may assist in AuNPs accumulation at the tumor site, active targeting of AuNPs is required to improve cancer treatment.

Therefore, Bo Ye et al. developed BSA and arginine-glycine-aspartic acid (RGD)-functionalized AuNRs, that were further internalized by neutrophils to obtain a neutrophil-based cancer cells delivery system, resulting in higher toxicity with laser irradiation in deeper tissues [92]. Other immune cells, the NK cells, are mainly responsible for inhibiting cancer cells because they can even recognize cells devoid of antibodies or cellular markers. Bin Liu et al. loaded CaCO_3_-coated Au nanostars together with Ce6 in the NK cells (AuNS@CaCO_3_/Ce6-NK). They showed prominent delivery of the nanoconjugates to the cancer cells with enhanced synergistic PTT, PDT, and immunotherapy [93]. The immune cell-mediated targeting is also known as “Hitchhiking” [94], and could be considered an effective targeting strategy without the use of any additional functionalizing agent.

### AuNPs for tumor starvation therapy

Although PTT has shown good therapeutic efficacy against cancer cells, hyperthermia or the penetration of NIR light is not sufficient to kill cells from deep tumor tissues. Moreover, hyperthermia induces the overexpression of HSPs in the cells, increasing the cell's heat tolerance ability. Thus, reducing the tumor-killing efficacy of PTT treatments. Since the synthesis of HSPs depends on ATP-supplied energy, the production of HSPs can be reduced or inhibited by restricting the energy supplies. Thus, PTT and glucose oxidase (GOx)-mediated cancer starvation therapy can work together as a multitherapeutic approach combining tumor growth reduction via consuming glucose and enhanced PTT effect via ATP depletion in tumor cells. Recently, Zhu et al. synthesized a nanoplatform based AuNRs and GOx coated by erythrocyte membrane for effective tumor targeting. They reported that these nano constructs could reach the tumor site without being recognized by the immune cells and enhanced the reduction in tumor growth by triggering the NIR-mediated PTT effect along with depletion of endogenous glucose to limit the energy supply to colon cancer cells [95]. Therefore, to enhance the anti-tumor efficacy of AuNPs, PTT can be combined with tumor starvation therapy by limiting the energy supplies to the cancer cells.

### AuNPs for imaging/diagnosis

In addition to cancer therapy, AuNPs acts as multipurpose tools for in vitro and in vivo cancer imaging/diagnosis [96]. This is because AuNPs have unique optical and electronic properties that render them properties for remarkable imaging [97] and diagnostic [98] agent. A combination of therapy and imaging can provide valuable information that can enable improved therapeutic efficacy and safety of AuNPs [99]. Various AuNPs-based multimodal imaging/diagnosis techniques are currently being explored, such as photoacoustic (PA) imaging, magnetic resonance imaging (MRI), and X-ray computed tomography (CT).

AuNPs can be used for PA real-time imaging because of their tunable optical absorbance in the NIR region that results from their SPR effect [100]. The PA imaging used an ultrasonic signal based on energy conversion from light to sound to analyze the structure and quality of tissue. The PA imaging and exogenous contrast agent can increase the resolution of subcellular images. Since the optical absorption of AuNPs is much higher than organic dyes due to their SPR effect, AuNPs can be used to obtain a highly visible contrast in the wavelength range of both the biological window, i.e., 650–1100 nm, and second near-infrared (NIR) spectral window, i.e., 1100–1350 nm. Thus, AuNPs-mediated PA imaging can facilitate the imaging of targeted areas that are deeply buried in the biological tissues and are hard to image using simple methods [101].

MRI is also a conventional anatomical imaging technique used to image soft tissues/cancers. However, MRI imaging has low specificity and can only detect tumors at late stages when they are millimeters in size [102]. The sensitivity of MRI is increased by using contrast agents, such as gadolinium (Gd^3+^) or manganese (Mn^2+^) because of their high electron magnetic moment. Though Gd^3+^ is the most widely used contrast agent, it is toxic in free form. Thus, it is administered in a stable chelate complex form. Recently, Gadolinium (Gd), an element used as a contrast agent for MRI images, was complexed with AuNRs to synthesize Gd/AuNRs nanocomplex. These nanocomplexes were capped with diacid polyethylene-glycol, loaded with doxorubicin (DOX), and investigated as a theranostic agent for pancreatic cancer. The MIA PaCa-2 cells internalized the Gd/AuNRs nanocomplex via the EPR effect, resulting in higher cytotoxicity towards MIA PaCa-2 than DOX alone. Thus, Gd/AuNRs nanocomplex also acted as a bio-imaging agent [103]. Thus, AuNPs-functionalized with imaging molecules can be used for theranostics applications, i.e., combined therapy and imaging purposes. However, active targeting of these Au-based nanoconstructs can specifically deliver the theranostic construct to the tumor site.

Nowadays, CT imaging accounts for 50–75% of imaging in the medical field. It creates cross-sectional 3D anatomical images of internal body structures with a high spatial and temporal resolution by utilizing high-energy electromagnetic radiation and a detector array [104]. However, CT imaging is not sensitive to soft tissue. Thus, AuNPs or iodine-containing probes are used as CT contrast agents to enhance the sensitivity of the CT imaging system [104]. However, compared to iodine, AuNPs is six times more efficient contrast agent. This is because AuNPs can produce contrast effects by absorbing higher X-rays due to their higher electron density [105]. Liu et al. showed that a combination of CT and MRI provides dual-mode imaging can diagnose cancer with more sensitivity and accuracy because it can integrate the advantage of both imaging systems [106]. Thus, a combination of more than one of the abovementioned imaging/diagnostic techniques in a single AuNPs construct can function as multimodal imaging/diagnostic agents, which is highly desirable and offers a more reliable, sensitive, and complete diagnosis.

### Functionalized AuNPs used for multimodal cancer therapy

The theranostic efficiency of AuNPs, i.e., conducting diagnosis and image-guided therapy is supported by the functionalization of AuNPs with targeting agents for cancer cells. Some of the recent and most-explored functionalization agents are discussed below (Table 1).

**Table 1:** List of various functionalizing agents used for AuNPs-based multimodal cancer therapy

Functionalizing agent	Composition	Cancer type	Size and Zeta potential	Type of therapy	Cell line/animal model	Result	References
EGF/HER-2/CD133 antibody	PLGA coated AuNSs	Breast	248.3 nm, − 14.7 mV	PTT imaging (808 nm laser (1 W/cm^2^) for 10 min)/USMR	SKBR3 cells and MDA-MB-231 cell line	Dual-modal molecular probe to provide US/MR contrast-enhanced imaging in vitro, as well as the targeted PTT effect of breast cancer cells induced by NIR-absorbed Au nanoshells	[109]
	Au nanocage	Breast	61.2 ± 4.85 nm, − 8.2 ± 1.25 mV	PTT (808 nm laser at 1 W/cm^2^, 5 min)	4T1cell/Female BALB/c mice	Significant improvement in the therapeutic effect of PTT by improved targeting efficiency and enhanced accumulation and uptake of nanoparticles in the cancer cells	[200]
	AuNPs functionalized with Ce6	Breast	21 nm	PDT (660 nm, 25 mW/cm^2^)	MDA-MB-468 and MCF 10A	PDT with Bifunctional EGF-Ce6 functionalized AuNPs efficiently induced cell death in TNBC cells by increasing ROS levels, while it did not affect normal cells	[51]
	AuNS@ICG-Ab	Breast	135.3 nm, − 31.5 mV	PTT(808 nm, 0.5 W/cm^2^, 3 min)/PDT (633 nm, 0.8 W/cm^2^, 5 min)/immunotherapy	CIK cells, SK-BR-3/ C57BL/6 and BALB/c nude mice	The AuNS@ICG-Ab-CIK can effectively diagnose and treat cancer	[39]
	AuNRs	Breast	55.1 ± 1.7 Length and 14.1 ± 1.1 nm diameter, 43.2 mV	Fluorescence imaging-guided (PDT 635 nm, 0.5 W/cm^2^, 2 min (/PTT 808 nm, 2.0 W/cm^2^, 5 min	MCF-7 cells /female BALB/c nude mice	MCF-7 cells could efficiently generate reactive oxygen species (ROS) and heat, and be more efficiently killed by a combination of PDT and PTT as compared with individual therapy	[186]
	anti-EGFR antibodies AuNRs	Lung	40 nm width and 148 nm length	PPT (854 nm, 1.5 W)/PA imaging	A549 cells	Anti-EGFR tagged AuNRs much larger accumulation as compared to untagged one. It was shown that the combination of pulse wave laser illumination of targeted nanoparticles produced a reduction of 93% ± 13% in the cell viability compared with control exposures, which demonstrates a possible application for minimally invasive therapies for lung cancer	[201]
	Nanobioconjugate AuNPs	Lung	63.91, − 14.7	PDT (673.2 nm, 10 J/cm^2^)	A549, CD133 + , CD44 + and CD56 + cells	AlPcS4Cl-AuNP-Ab nanobioconjugate (NBC) are biocompatible and NBC photodynamic effect induces the preferred cell death, but it also shows enhanced and effective lung CSC destruction	[53]
	Au nanostars	Prostate	120 nm, − 22.47 mV	PTT((808 nm, 0.8 W/cm^2^, 5 min/PDT (NIR-light irradiation, 7 min)/CTX	PC3 cell-line/ male BALB/c athymic nude mice	Au nanostars@IR820/DTX-CD133 for in vitro and in vivo therapy achieves the excellent antitumor effects of the synergistic PTT/PDT/CT strategies under the NIR-light irradiation	[155]
Serum albumin (SA)	BSA-AuNCs	Breast	–	PDT (λ = 405 nm, 66 mW/cm^2^)	MCF-7 and MDA-MB-23	BSA-AuNCs can induce efficient cytotoxicity	[202]
	PLGA surface modified AuNRs	Colon	245.8 nm, − 8.6 mV	PTT (808 nm, 1.5) for 4 min) /CTX	CT26, and MCF7	The HADP NPs showed promising combined PTT- and chemotherapeutic effects without inducing undesired side effects on a murine colon cancer animal model	[203]
	BSA modified AuNR	Lung	50 nm, + 35 mV	PTT(808,1 W/cm^2^ for 8 min)	Lewis cells/female C57BL/6 mice	Excellent biosafety of AuNRBR/N without laser irradiation, and exhibited superior therapeutic effect on Lewis tumor due to the optimal tumor targeting of neutrophils and multistage delivery of AuNRBR for deep tumor diffusion, which also improved the survival rate of mice	[92]
	AuNPs	Liver	49 nm	PTT (2 W, 808 nm, 30 min)	HepG2 and HepB5 cells	Alb-AuNPs showed no cytotoxic effect, Alb-functionalized AuNPs leads to increased intracellular uptake in liver cancer cells by selective targeting of Gp60 receptors	[112]
Glutathione	Hyaluronic acid functionalized AuNRs	Breast	Length 55.1 ± 1.7 and diameter 14.1 ± 1.1 nm, 43.2 mV	Fluorescence imaging-guided (PDT 635 nm, 0.5 W/cm^2^, 2 min (/PTT 808 nm, 2.0 W/cm^2^, 5 min	MCF-7 cells /female BALB/c nude mice	MCF-7 cells could efficiently generate reactive oxygen species (ROS) and heat, and be more efficiently killed by a combination of PDT and PTT as compared with individual therapy	[186]
	Glutathione corona coated AuNPs	Liver	Aggregated AuNP, 239 ± 73 nm and 254 ± 64 nm, − 33 mV	PTT (760 nm, 1.26 s)	Hep G2 line	a-DG-AuNPs readily internalized in HeP G2 cells, efficient cancer cell ablation occurs via two-photons excitation PTT	[122]
Lactoferrin (LF)	AuNRs	Liver	70 nm in length and 11.5 nm in width, − 15 mV	PTT (980 nm, 0.5 W/cm^2^)	HepG2 cells/nude mice	Surface coated and medium size AuNRs shown enhanced uptake and retention by cancer cell, AuNR70@PEG-LF shown completely destroyed the tumors without recurrence after one single treatment achieved due to synergistically via proper size and ligand conjugation	[124]
	Lactoferrin-conjugated AuNP	Brain	5 nm	PTT (532 nm NIR diode laser (4 W/cm^2^) for 5 min	Human GBM U87MG cell line/Male BALB/c nude mice	Orally administered Lf-PEG-AuNP exhibit an outstanding temperature rise in GBM and significantly reduce tumor volume under laser irradiation	[129]
Folic acid (FA)	Si coated AuNPs	Breast	25 nm, − 19.7 mV	PTT (810 nm, 185 mW, 139 s)	MCF-7 and MDA-MB-231 cells	MTX-FA loaded Au@SiO_2_NPs had shown improved the efficacy of laser therapy in breast cancer cell destruction	[47]
	AuNPs	Brain	10 ± 2 nm	PTT (808 nm, 0.8 W/cm^2^)	C6 glioma	Photoresponsive Au-decorated polymer nanoparticles (FA-PGPNPs) are promising nanoprobes with targeting ability, enhanced tumor PDT, cell tracking, and PTT effect	[131]
Peptides	Mesoporous silica-coated gold cube	Breast	116.5 nm, 24.5 ± 1.6 mV to 5.6 ± 0.5 mV	PDT ( 635 nm)/PTT (808 nm)/multimodal bioimaging	4T1 and L929 cell line/ nude mice	Showed high therapeutic performance and multiplexed imaging, which provides an innovative paradigm for targeted tumor therapy	[204]
	AuNPs	Colon	56.1 ± 0.3 and 62.8 ± 0.4 nm, − 30.1 ± 1.7 and − 20.8 ± 1.3 mV	PTT (808 nm laser (2 W/cm^2^) for 5 min)/PA	HUVEC and HCT-116 cell line/female nude mice	The Au-RRVR nanoparticles could form large aggregates within tumorous tissue resulting in improved tumor accumulation and retention, which can further activate the PA and PTT effect of AuNPs for sensitive imaging and efficient therapy of tumors	[205]
	Mesoporous silica coted AuNPs	Lung	20 nm	PTT (808 nm laser, 1.2, 0.9, 0.6, and 0.3 W/cm^2^ for 5 min)/CTX	A549, HOB, and HBMSC cells/mice	Au@MOF@MS-ICG-dYNH-PAA (AMMD) shows enhanced cellular uptake on tumor cells. Benefiting from this ultra-high affinity to tumor cells and the photothermal effect of ICG, the dual-drug-loaded AMMD (BCAMMD) modified with ICG exhibits superior therapeutic efficacy on spinal tumors	[206]
	Polypeptide-modified AuNCs	Lung	85.2 nm, − 17.44 ± 2.48	PDT (633 nm, 100 mW/cm^2^ for 5 min)/CTX/fluorescence imaging	A549 cells, Female BALB/c-nude mice	Nanoprobes could be efficiently internalized into A549 cells and then significantly enhance the mortality of cancer cells compared with free Ce6 and DOX, and shown excellent tumor targeting ability, long blood circulation time, and could remarkably inhibit the growth of tumor	[29]
	(PD-L1) peptides modified gold nanoprisms	Lung	3.41 ± 5.22 mV	PTT (633 nm, 0.8 W/cm, 1 min to 10 min)/PDT(633 nm (0.8 W/cm^2^, 10 min)/PA imaging	HCC827 and A549 cells/female BALB/c nude mice	AuNPs@PEG/Ce6-P are biocompatible and significantly suppress tumor growth through PTT and PDT from AuNPs and Ce6, respectively	[138]
	AuNPs	Liver	72.4 nm, − 12.5 ± 2.1 mV	PTT (0.8 W/cm^2^, 10 min, 1 min/PDT (808 nm, 6 min)	HCC-LM3 cells/nude mice	Excellent biocompatibility, high absorption by cancer cells, enhanced PDT/ PTT combination therapy under laser irradiation, significant inhibition of tumor growth	[56]
	Au nanostars	Lung	80 nm, − 10.1 mV	PTT/PDT/PA	A549 cancer/ Female BALB/C nude mice	Au nanostars@BSA/I-MMP2 exhibited excellent stability and biocompatibility and effectively internalized by A549 cancer cells and exhibited remarkable antitumor efficacy	[116]
	AuCNs	Pancreatic	53 nm, − 17 and − 10 mV	PTT (750 nm, 2 W/cm^2^,5 min)/PDT (652 nm laser (50 m W/cm^2^, 5 min)	PANC1-CTSE/mice	The AuS-U11 represents a very promising imaging-guided PDT/PTT therapeutic agent for pancreatic tumor therapy, along with highly synergistic therapeutic effects against pancreatic tumors as well as the reduced side effects in normal pancreas tissue	[55]
	AuNPs	Prostate	13 nm after adding Serum alkaline phosphatase (ALP) AuNPs aggregated to 500 nm	PTT (650 nm, 5/cm^2^, 10 min	PC-3 cells, MCF-7/ male BALB/c nude mice	The AuNPs@Peptide can be enzymatically assembled into large aggregates and enhance the temperature of the tumor during PTT	[207]
	Au nanostars	Prostate	82.5 ± 6.5 nm in diameter with sharp edges, − 3.74 ± 0.09 mV	PTT (808 nm laser (optical density 2.5 W/cm^2^, 3 min)	PC-3, DU145, LNCaP, 3T3 cells/ nude mice	These nanostars have excellent light-thermal conversion efficiency in the NIR region, biocompatible surface and strong cellular penetration. MSCs loaded with the TAT-conjugated Au nanostars (TAT-Au nanostars) could facilitate the assembly of the nanostars in the lysosomes inside MSCs as an “engineering factory” and excrete the microvesicles loaded with Au nanostars for tumor recognition and distribution	[85]
	AuNRs	Prostate	52.33 ± 8.05 nm length, 13.99 ± 1.09 nm width, − 21.6 mV	PTT (808 nm, 0 to 5 W,/PDT (laser irradiated 2.5 and 5 W/cm^2^	PC-3 cells,/ Balb/C nude mice	In vitro study with the castration resistance prostate cancer cell exhibited a significant PTT effect as well as enhanced thermodynamic therapy via generating free radicals. P-p38 and p-JNK proteins, as key proteins involved in the cells’ stress responses, were found to be upregulated by the synergetic treatment	[148]
Aptamers	Deoxyribonucleic acids-gold particle	Breast	13 nm	PTT/PDT (660 nm, 0.8 W/cm^2^ for 5 min)/in situ imaging-guided/CTX, gene therapy	MCF-7 cells, HeLa cells, L02 cells/ female nude mice	The Apt-DNA-Au nanomachine provides superior in vitro and in vivo sensitivity and specificity of the Apt-DNA-Au nanomachine for TK1 mRNA have achieved real-time monitoring of the dynamic change in tumors during therapy	[179]
	AuNPs modified with AS1411 and DNA	Colon	24.42 nm, − 35.8 mV	PTT(808 nm, 1 or 2 W/cm^2^ for 5,10,30, 60,120 min)/CTX	SW480 cells	The AS1411 NPs exhibited the most efficient cytotoxicity and markedly enhanced inhibition effect on cells proliferation to SW480 cells under laser exposure when compared to the NPs merely with PTT or chemotherapy	[117]
	AS1411 aptamer modified Au nanocage	Lung	10 nm	PTT (808 nm 1 W/cm^2^ for 5 min), /CTX/genetic	NCI-H889/ BALB/c nude mice	The combined genetic, chemotherapeutic, and PTT treatment group exhibited more than 90% tumor inhibition ratio (tumor signal) and a ~ 67% survival rate compared with a 30% tumor inhibition ratio and a 0% survival rate in the passive genetic treatment group	[177]
	Aptamer-modified HAu nanoshells	Lung	10–20 nm to 100–200 nm	PTT (808 nm, for 5 min)	MRC-5, MCF-10A, A549, MCF-7	Aptamer-modified nanoparticles were accumulated selectively in tumor cells (A549, MCF-7) and this fact contributed to the reduction of tumor spheroids viability and size	[208]

#### Epidermal growth factor (EGF)/human epidermal growth factor 2 (HER2) antibodies

The Epidermal Growth Factor (EGF) receptor is highly present in cancer cells and is selective for its ligand, EGF. The EGF is a small secretory protein responsible for tumor growth and proliferation. Reports indicate the use of EGF for selective uptake of nanoparticles by the cancer cells. In a study, EGF-conjugated AuNPs showed uptake of 63 nanoconstructs per minute by MDA-MB-468 triple-negative breast cancer cells. These EGF-AuNPs nanoconjugates were also combined with Ce6 for the combined PDT effect. It was found that these nanoconstructs induced apoptosis in 38% of cancer cells and necrosis in 58% of cancer cells at 660 nm, 25 mW/cm^2^ irradiation. Moreover, the nanoconstructs-treated cancer cells showed nine times higher ROS content than normal cells [51].

Breast cancer is the most common type of cancer in women with high intra-tumoral heterogeneity, resulting in varied therapeutic responses due to wide-ranging phenotypes and morphological profiles [51]. Human epidermal growth factor receptor 2 (HER2), a member of the EGF receptor family, serves as a biomarker, especially for breast cancer and gastric cancer [107, 108]. Liang et al. demonstrated that sharp-edged Au nanostars conjugated with HER2 monoclonal antibodies improved tumor targeting and retention in the SK-BR-3 human breast cancer cell line [39]. Qi Dong et al. used AuNSs-poly (lactic-co-glycolic acid) (PLGA) magnetic hybrid nanoconstructs conjugated with anti-Her2 antibodies for dual-modal ultrasound/MRI and PTT effect on SK-BR-3 breast cancer cells when irradiated using 808 nm laser at 1 W/cm^2^ for 10 min [109]. HER2-targeted AuNPs conjugated with Trastuzumab (HER-2 monoclonal antibody) also showed promising therapeutic results against gastric cancer [110]. Although HER2- has improved the clinical outcome in breast and gastric cancers, it showed poor outcomes in other cancers [108]. Therefore, HER2-related studies, alone or in combination, are under investigation, and it is possible to obtain more diverse results in the near future.

#### Serum albumin (SA)

The serum albumin (SA) serves as efficient drug delivery and tumor-targeted vehicle because it can conjugate or encapsulate chemotherapeutic agents [111]. The SA nanovesicles can accumulate in tumor tissue due to their interaction with the gp60 receptor that is overexpressed in various tumors. Moreover, nanoconstructs with albumin can rapidly internalize in the cancer cells via caveolae-mediated endocytosis with the help of a glycoprotein known as a secreted protein, acidic and rich in cysteine. It was found that the albumin-AuNPs nanoconstructs can internalize liver cancer cells via gp60 receptor targeting [112]. Moreover, when HepG2 or hepatocytes cells were treated with albumin-AuNPs nanoconstructs and then irradiated using a 2 W, 808 nm laser, higher apoptotic and necrotic rates were observed in HepG2 cells than normal hepatocyte cells, indicating selective therapeutic efficacy [112]. Liver cancer is a worldwide health challenge. It is estimated that there will be > 1 million cases by 2025 [113]. Although surgical resection is a possible therapy for liver cancer, it is feasible in only < 30% of patients. Since other treatment strategies show modest results in the treatment of liver cancer, new therapeutic approaches are still needed [112, 114]. Thus, AuNPs-based therapies could be a possible alternative to conventional treatments to treat liver cancer.

The SA functionalized AuNPs have also been used to treat colon cancer which is the second most commonly diagnosed cancer in women and the third most diagnosed cancer in men. Colorectal cancer has a high mortality rate globally because the conventional treatments may induce drug resistance and lack selectivity [95]. It has been reported that the clusters of AuNPs, i.e., AuNCs, of ~ 88 nm size consisting of albumin-AuNPs (~ 4.5 nm) (AuNCs/BSA-AuNPs) showed significant hyperthermia effect in the HCT116 colon cancer mice model after laser irradiation (1.5 W/cm^2^, 10 min), suppressing tumor growth [115]. Moreover, when AuNCs/BSA-AuNPs were further modified with cy5.5, they showed good fluorescence-based optical visualization in the HCT116 colon cancer mice model, suggesting efficient tumor targeting [115]. The AuNCs show a red-shift phenomenon, strengthening NIR absorption in the wide range (650–950 nm), displaying a high anti-tumor effect due to high hyperthermal conversion compared to AuNPs. However, AuNCs with larger AuNPs lose their inherent fluorescence and cannot diagnose or detect tumors, though they show a significant hyperthermic effect. Therefore, AuNCs with small-sized AuNPs might be suitable for both detection and PTT effect against various types of cancers.

**Fig. 2:**
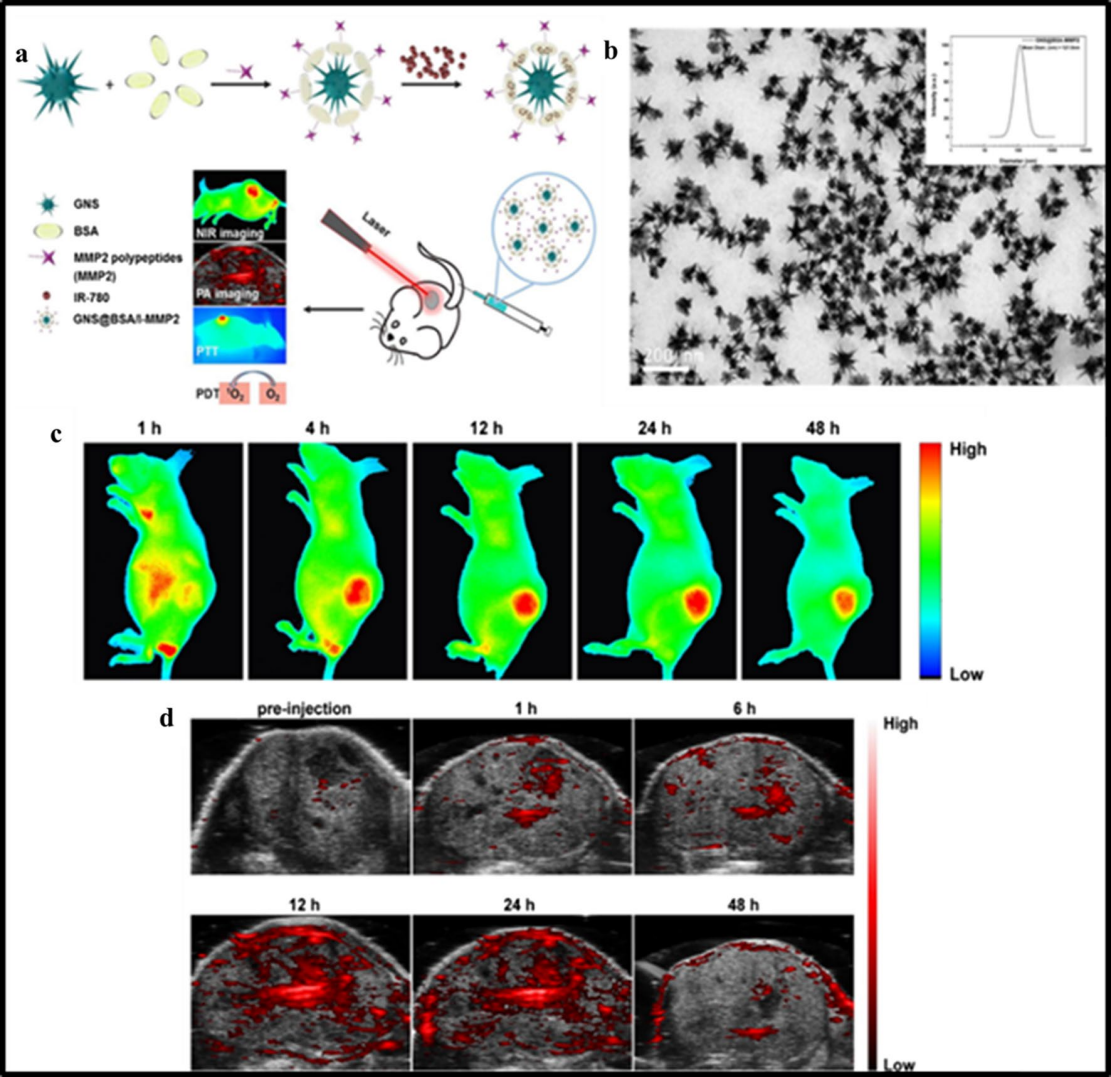
**a** Schematic illustration of the synthetic procedure of AuNS@BSA/I-MMP2 NPs and their applications, **b** TEM image of AuNS@BSA/I-MMP2 NPs, **c** in vivo NIR fluorescence images of mice bearing A549 tumors after injection of AuNS@BSA/I-MMP2 NPs (excitation = 710 nm, emission = 790 nm), **d** PA images of AuNS@BSA/I-MMP2 NPs treated mice at different time intervals (excitation = 780 nm). Reproduced with permission from [116]. Copyright ©2019, Elsevier

A study showed that MMP antibodies could be conjugated with albumin-coated Au nanostars as carriers of IR-780 for efficient lung tumor diagnosis and therapy (Fig. 2) [116]. Therefore, a combination of targeted multifunctional AuNPs-mediated colon cancer therapy suggests synergistically enhanced anti-cancer effect and reduced systemic toxicity towards normal cells [117, 118].

#### Glutathione

It has been observed that functionalizing the surface of AuNPs with glutathione can control the cell internalization of AuNPs without disrupting the cell membrane. It is because the negatively charged glutathione-AuNPs are adsorbed onto the cell membrane [119]. In addition, glutathione-capped AuNPs can exhibit photoluminescence and size-independent light emission at 600 nm and 800 nm at 396 nm and 350 nm excitation wavelength, respectively [120]. Steckiewicz et al. showed that glutathione stabilized AuNPs and conjugated with cytarabine induce more cell death than cytarabine alone on various cancer cells [121]. Buonerba et al. showed that the salt-induced well-defined-sized aggregates of both glutathione-AuNPs (239 ± 73 nm) and AuNPs coated with glutathione functionalized with dansyl fluorophore nanoparticles (254 ± 64 nm) were efficiently internalized in human hepatocytes HepG2 cell lines via endocytosis without inducing cytotoxicity. After internalization, the aggregates of glutathione-AuNPs and dansyl fluorophore glutathione-AuNPs produce dispersed spherical nanoparticles in the cytoplasm that rapidly crosses the nuclear membrane. However, they found that the PTT ablation in dansyl fluorophore glutathione-AuNPs aggregates-treated cells was higher than that of glutathione-AuNPs aggregates-treated cells after NIR irradiation with pulsed lasers tuned at 760 nm for 1.26 s [122]. This is possibly because the chromophore acts as both an antenna and transducer of the NIR radiation. Thus, AuNPs coated with biocompatible glutathione are widely studied as a drug delivery system because glutathione provides a stealth effect against serum proteins and renders glutathione-AuNPs highly resistant to adsorption by serum proteins [119, 123].

#### Lactoferrin (Lf)

In general, tumor cells overexpress lactoferrin receptors (LfRs) to fulfill their requirements. In a study, a series of AuNRs with a tunable dimension of similar aspect ratio with similar photothermal transfer efficiency were surface-modified with PEG and covalently conjugated with tumor-targeting ligand lactoferrin (Lf). The study showed that these AuNRs (70 nm in length and 11.5 nm in width) exhibited photothermal cytotoxicity in HepG2 liver cancer cells when irradiated with 980 nm diode laser 0.5 W/cm^2^ power. Further, the HepG2 xenograft nude mice model showed that the AuNR70@PEG-Lf showed the highest tumor accumulation and prolonged circulation time due to the synergetic effect of dimension and surface coating. These xenograft models showed a reduction in tumor volume after NIR irradiation (980 nm and 0.5 W/cm^2^ power for 3 min), suggesting the PTT potential of AuNR70@PEG-Lf against liver cancer in the NIR-II window [124].

Since LfRs are highly expressed in the intestine, blood–brain barrier, and cancer cells, targeting AuNPs via Lf is proposed to be an effective strategy. Glioblastoma (GBM), a form of malignant central nervous system tumor, has a high incidence and mortality rate [125] and high reoccurrence chances [126]. Although surgery, followed by focal RT, laser interstitial thermal therapy, and adjuvant CTX, is the most prevalent treatment for glioblastoma, the delicate anatomical structure of the brain reduces the chances of successful surgery [127]. Moreover, GBM patients have shown an average two-year survival rate with RT and temozolomide so far, possibly due to the resistance developed in patients against RT. Thus, there is a need to develop alternative approaches for effective GBM treatment by improving intrinsic RT resistance [128]. AuNPs with < 100 nm diameter can generally cross the blood–brain barrier (BBB) due to leaky vasculature, resulting in their accumulations in the tumor tissue. Thus, demonstrating a significant therapeutic PTT effect against glioblastoma. However, enhanced blood stability and half-life of AuNPs in the blood and targeted delivery are also critical criteria that should be considered for the successful delivery of AuNPs to the brain tumor site. Therefore, AuNPs-mediated PTT should be combined with targeted delivery for effective GBM treatment. Kim et al. suggested that AuNPs-conjugated with Lf can reach GBM in the brain via oral absorption [129]. In addition, they used glutathione and PEG to enhance the blood circulation time of Lf-AuNPs. After oral administration, they observed 11-fold and eightfold higher AuNPs concentrations in blood and GBM, respectively [129]. Further, they found that laser irradiation post-Lf-AuNPs delivery can increase the temperature in GBM, resulting in tumor volume reduction. Thus Lf can be used as an efficient targeting molecule to GBM across the blood–brain barrier via the oral route.

#### Folic acid (FA)

Folic acid (FA), vitamin B9, is another tumor cell targeting agent that binds to folate receptors and facilitates intracellular uptake via endocytosis [130]. The folate receptors are absent in healthy non-proliferating cells while overexpressed in proliferating cancerous cells. It was observed that FA-conjugated N-(2-hydroxy)propyl-3-trimethylammonium chitosan chloride (HTCC)-stabilized AuNPs were more internalized by Caco-2, HepG2, and HeLa cancer cells than AuNPs. Moreover, it was found that the surface modification of Au-decorated acrylic copolymeric nanoparticles with FA improved the targeting efficiency of NPs by 71.8% cell compared to 28.8% uptake for the non-conjugated NPs, resulting in increased PTT effect on glioma cells under near-IR irradiation at 808 nm [131]. Similarly, FA-conjugated poly(ethylene glycol) coated Au@iron oxide core–shell nanoparticles decreased the growth of KB cancer cells by ~ 62% and MCF-7 breast cancer cells by ~ 33% (28994325). These studies suggest that FA-conjugated AuNPs showed an enhanced therapeutic effect on various types of cancer cells compared to bare AuNPs.

Kumar et al. showed the higher affinity of FA-conjugated AuNPs towards folate receptor-positive MCF7 breast cancer cells than folate receptor-negative A549 cancer cells [132]. In accordance, another study also showed higher induction of apoptosis by FA-targeted Fe_2_O_3_@Au than non-targeted Fe_2_O_3_@AuNPs in human nasopharyngeal (KB) cancer cells [133]. Thus, suggesting FA as a promising targeting ligand for folate receptor-positive cancer cells.

#### Programmed death-ligand 1 (PD-L1) peptides/antibodies

The PD-L1, a type 1 transmembrane protein, is highly-expressed on various cancer cells, including breast cancer, lung cancer, colorectal cancer, and melanoma, and has been implicated as a biomarker for cancer [134]. The PD-L1 overexpression is associated with cancer growth [135]. The PD-L1 blocking antibodies, such as MEDI4736 and MPDL3280A, are currently approved for cancer therapy [136]. It has been found that PD-L1 antibodies conjugated AuNPs significantly decreased the growth of oral squamous carcinoma cell line (SCC-25) via increasing the expression of apoptotic proteins but did not affect the growth of normal HaCaT keratinocytes cells [137]. Thus, indicating the expression of PD-L1 on cancer cells. Bin Liu et al. developed a nanoplatform, i.e., AuNPs@PEG/Ce6-P, by conjugating PEG-coated AuNPrs with Ce6 and human programmed death-ligand 1 (PD-L1) peptides to target lung tumor cells for imaging-guided and actively PTT/PDT. The AuNPs@PEG/Ce6-P nanoplatform demonstrated a remarkable affinity to HCC827 lung cancer cells with high PD-L1 expression, resulting in tumor growth suppression due to synergistic PTT/PDT effect. In addition, with the help of this nanoparticle system, real-time visualization via fluorescence and PA imaging was also possible [138]. Lung cancer contributes to about 20% of cancer-related mortalities worldwide, possibly due to high chances of relapse associated with self-renewal CSCs resistant to conventional cancer treatment [139, 140]. As observed, PD-L1 targeted AuNPs@PEG/Ce6-P nanoplatform showed remarkable targeting ability, dual-mode imaging, and promising anticancer potential owing to enhanced PDT/PTT effect on lung cancer. Thus, suggesting that peptides targeted to PD-L1 are effective targeting agents for AuNPs-based cancer therapy.

It has been known that combination therapies enhance the therapeutic efficiencies. In accordance, it has been found that the combinational nanoconstructs comprising of DOX and AuNPs-conjugated with anti-PD-L1 antibodies showed significant intracellular uptake of DOX. Moreover, post-NIR irradiation, these nanoconstructs showed synergistic suppression of colorectal CT-26 cancer cells proliferation via increased cell cycle arrest and apoptosis [141]. Thus, anti-PD-L1 antibodies/peptides can be used to target various cancer cells for multiple therapeutic strategies.

#### RGD (Arg-Gly-Asp) peptides

The RGD (Arg-Gly-Asp) peptides are associated with several types of integrins that are heterodimer cell surface receptors that are highly expressed in cancer cells and are involved in the adhesion of cells to the extracellular matrix [142]. Integrins are involved in the signaling pathways responsible for cancer growth and metastasis. The αvβ3 integrins bind with the RGD peptide in extracellular matrix proteins, such as fibronectin [143]. It has been found that the RGD sequence enables the internalization of AuNPs into the tumor cells via endocytosis and localize in the late endosomes and lysosomes of breast cancer cells [144]. It has also been observed that due to the specific targeting, the RGD-labelled AuNPs can be metabolized and cleared out of the body, indicating a good biosafety profile [106].

Recently, Hua et al. successfully constructed a nanoplatform for cancer theranostic by using cyclic RGD (cRGD) peptide-modified Au-iron oxide nanoparticle (Au4-IO NP-cRGD) for enhanced MRI dual-modal imaging-guided Fenton reaction-assisted radiotherapy and showed 81.1% tumor-suppression in vivo [70]. The Au nanostars can also be labeled with Raman molecules and RGD peptides for A549 human lung adenocarcinoma cells-targeted SERS-imaging and image-guided PTT in both the NIR-I and NIR-II windows [145]. Albertini et al. showed that the RGD-conjugated AuNPs have also shown enhanced accumulation in the brain of intracranial tumor models compared to bare AuNPs, possibly due to the presence of αvβ3 integrin receptors on the blood–brain barrier [146]. These studies suggest that RGD could be used for targeting AuNPs to glioblastomas.

#### Other peptide/antibodies/aptamers

Various other peptides, antibodies, and/or aptamers are used to deliver AuNPs to cancer cells. Bombesin (BBN) is a peptide targeting gastrin-releasing peptide receptors that are highly expressed in various cancers, such as lung, breast, and prostate cancers [147]. In a study, BBN peptide-tagged m-SiO_2_ coated AuNPs can specifically target the NPs to prostate cancer cells overexpressing gastrin-releasing peptide receptors [148]. Prostate cancer ranks fifth, about 6.6%, among cancer-related mortalities in men worldwide [149–151]. Whole-gland treatments, such as CTX, RT, and castration therapy, are often prescribed to men with prostate cancer at early stages, which have adverse effects [152]. However, in some cases, castration therapy becomes ineffective due to the emergence of castrate-resistant prostate cancer (CRPC) that induces resistance to CTX and RT, thus, shortening the survival time of prostate cancer patients [153, 154]. Hence, exploring a highly effective and well-tolerated therapeutic method for CRPC patients is needed. A multifunctional nanoplatform composed of IR820-loaded Au nanostars with the guided effect of CD133 antibody also showed image-guided/targeted synergistic PTT/PDT/CTX effects to treat castration-resistant prostate cancer (CRPC) [155]. When combined with targeting agents, such as peptides and/or antibodies, AuNPs can be used to treat CRPC.

Another peptide, U11, has also been explored for targeting AuNPs. In a study, the affinity and cellular uptake of nanoconstructs consisting of AuNCs, 5-ALA, and cyanine dye Cy5.5 (a CTSE-sensitive imaging agent) by pancreatic cancer cells were increased by labeling the nanoconstructs with U11 peptide, a ligand for urokinase-type plasminogen activator receptor (uPAR) [55]. With minimal side effects, these nanoclusters showed significant therapeutic efficacy with endomicroscopy-guided PTT/PDT [55]. Pancreatic cancer ranks fourth in cancer-related mortality cases in the US [156]. This is possible because only 20–30% of patients with pancreatic cancer respond well to gemcitabine (GEM)-based CTX due to chemoresistance caused by the presence of high interstitial fluid pressure and dense tumor stroma. Moreover, curative surgical resection is not advisable for most pancreatic cancer patients [157]. Since PTT can improve the therapeutic efficacy of chemotherapeutic drugs, AuNPs under NIR irradiation and chemotherapeutic drugs are being studied for pancreatic cancer treatment [158–161]. In addition, when combined with targeting ligand, AuNPs showed enhanced therapeutic effects against pancreatic cancer [55].

Studies on AuNPs attached with PSMA (prostate-specific membrane antigen)-specific targeting ligand have increased rapidly. PSMA is an integral membrane glycoprotein that is overexpressed only in androgen-independent prostate cancers. A dual aptamer, i.e., anti-PSMA RNA aptamer (A10) and a peptide aptamer (DUP-1), conjugated Au nanostars were developed for PSMA(+) and PSMA(−) cells, respectively, which were highly efficient in photothermolysis NIR laser (at 808 nm and 0.3 W/cm^2^) [162]. Similarly, it was also observed that PSMA-positive PC3pip prostate cancer cells had higher PSMA-1-conjugated AuNPs uptake than PSMA-negative PC3flu prostate cancer cells [163]. Thus, the functionalization of AuNPs with cancer-specific aptamers could be an effective targeting strategy.

#### Functionalized AuNPs as a carrier for nucleic acids

The nucleic acid [such as small interference RNAs (siRNAs) and microRNAs (miRNAs)]-based therapies have revolutionized anti-cancer research studies by regulating signaling pathways responsible for cellular growth and differentiation [164]. Nevertheless, the degradation of nucleic acids in the physiological conditions and the intrinsic negative charge of nucleic acids are the significant hurdles limiting their entry into the cells [165]. This generates a need for delivery systems to deliver nucleic acids that protect them from physiological conditions. Due to the unique properties and biocompatibility, AuNPs are used to deliver nucleic acids to the target cells without any transfection agent [166]. Moreover, cationic AuNPs can form electrostatic complexes with nucleic acids, rendering nuclease protection to nucleic acids and efficiently delivering it to target cells [167]. It has been found that AuNPs can also act as a carrier for the delivery of siRNA [168] and miRNA [169] to down-regulate the expression of PD-L1 and Sp1, respectively, in lung cancer and can also provide additional PTT effects against lung cancer.

Furthermore, functional modification of AuNPs with cationic carbosilane dendrons containing a thiol moiety can stabilize the AuNPs, providing both hard metal core and soft surface dendrons to the nanoparticles. The metal core of the nanoconstructs can help AuNPs to accumulate more efficiently at the tumor site, enhancing the cellular uptake by the EPR effect. In contrast, the soft dendrons enable the AuNPs to bind with nucleic acid. Such gold nanocomplexes showed efficient delivery of siRNAs into the cells [170]. Despite using AuNPs alone, Au-dendrimers nanohybrids are also efficiently used to deliver nucleic acids for cancer gene therapy [171].

The siRNA-conjugated AuNPs also exhibited a combination of radiotherapy along with gene therapy [172, 173]. Similarly, AuNPs can co-deliver DOX and siRNA for combinational CTX and gene therapy [174, 175]. Moreover, modifications of nucleic acid-AuNPs conjugates with active targeting agents can further enhance their selectivity for tumor cells [169, 176]. Yang et al. developed a siRNA and DOX delivery system composed of Au nanocages functionalized with AS1411, an aptamer for nucleolin receptors, for site-specific targeted delivery to the tumor cells [177]. They also used double-stranded DNA (dsDNA) as the rigid support for Au nanocage and MMP-2 cleavable peptide that facilitates the destruction of Au nanocages by MMP-2 enzyme that are overexpressed in the tumor microenvironment, resulting in multifunctional and tumor-specific gene therapy, CTX, and PTT [177]. AS1411-modified AuNPs have been well-studied for active cancer cells targeting [117]. Thus, these studies suggest the prospect of multifactorial therapy due to its intrinsic therapeutic properties and its ability to act as nanocarriers for gene therapy and CTX. Further, attachment of targeting agents can enhance their tumor cell-specific cellular uptake and enhance therapeutic efficacy.

### Stimuli-responsive nanoconstructs for cancer theranostic

Nowadays, multi-modal theranostics is considered a promising approach for cancer treatment and imaging [178, 179]. Stimuli-responsive drug delivery systems showed advantages in controlling the drug release in response to exogenous stimuli (such as light, temperature, electric pulses, magnetic field, and ultrasound) or endogenous stimuli (such as enzyme, pH, and redox) [180, 181]. Among these AuNPs-based nanosystems, light-sensitive (NIR light) nanoconstructs are mostly preferred. Firstly, because AuNPs can absorb NIR light radiation and convert them into heat through the SPR effect. Secondly, cancer cells are more sensitive to heat than normal cells because of the poor vascular structure of tumor tissues. Thus, avoiding side effects on the normal cells. The hyperthermia is further associated with thermo-responsive nanosystems for the controlled release of drugs [182].

**Fig. 3:**
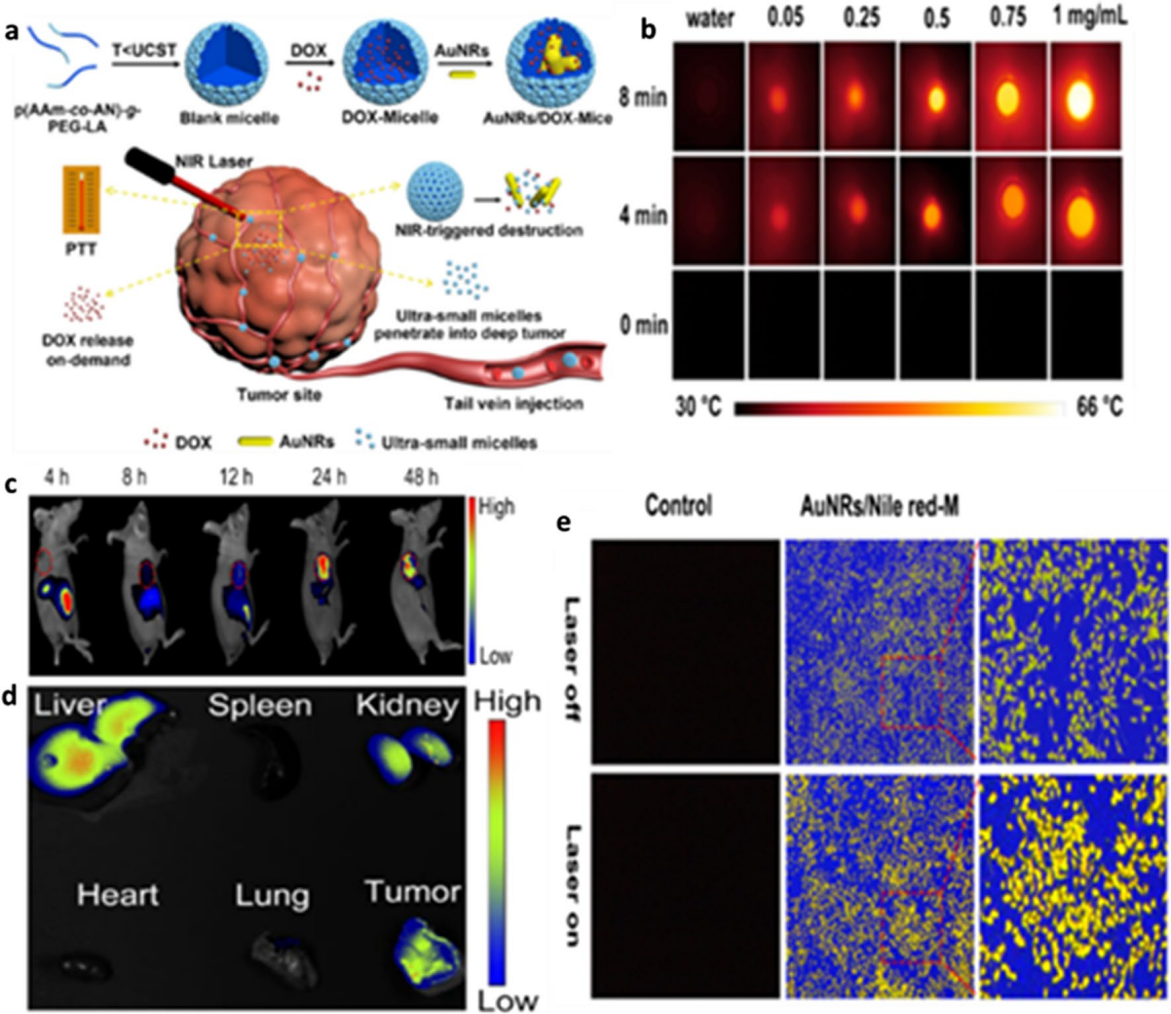
**a** Schematic representation of the developed size-shrinkable p(AAm-co-AN)-g-PEG-LA nanomicelles loaded with AuNRs and DOX, **b** infrared thermal images of AuNRs-micelle after laser irradiation, **c** In vivo real-time fluorescence images, **d** Ex-vivo fluorescence image of the excised organs and tumor of HepG2 tumor-bearing mouse after *i.v.* injection of AuNRs/ICG micelle, and **e** Fluorescence images of HepG2 cells treated with AuNRs/Nile red-M. Reproduced with permission from [183]. Copyright ©2021, ACS Publication

Danju Wu et al. reported the synthesis of a size-shrinkable thermo-responsive nanomicelle system composed of copolymer poly(acrylamide-acrylonitrile)-polyethylene glycol-lipoic acid (p(AAm-co-AN)-g-PEG-LA) with an upper critical solution temperature (UCST) behavior. These nanomicelles were loaded with AuNRs and DOX, an anti-cancer drug. They showed that these nanomicelles rods of the 54 nm length and 14 nm width were initially accumulated on the tumor periphery via the EPR effect. After NIR irradiation (λ = 808 nm, 2 W/cm^2^, 8 min), the AuNRs produce heat leading to PTT ablation of tumor tissue (Fig. 3). In addition, the increase in temperature results in the breakdown of nanomicelles into ultra-small nanomicelles (approx. 7 nm) that facilitates the penetration of ultra-small nanomicelles into the deep tumor site for the smart delivery of the loaded drug [183].

**Fig. 4:**
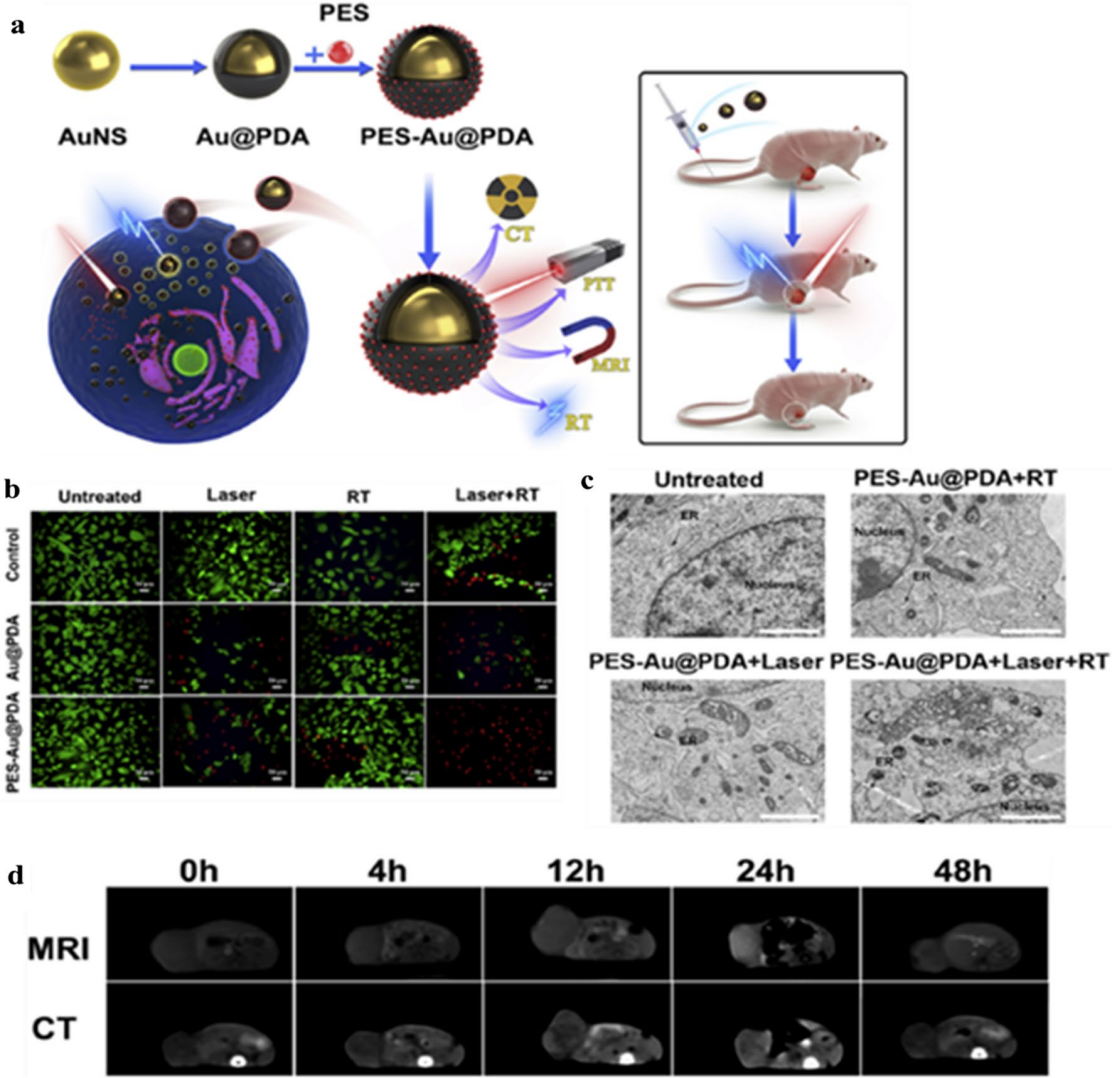
**a** Schematic representation of PES-Au@PDA to achieve synergistic PTT and RT of glioblastoma cancer cells, **b** Confocal laser scanning microscopy images of SW1783 cells stained with calcein AM (green) and propidium iodide (PI) (red), **c** ER structures of SW1783 cells, **d** CT and T1-weighted MR images were acquired at the indicated times (0, 4, 12, 24, 48 h) following intravenous injection of 12 nm/kg PES-Au@PDA NPs. Reproduced with permission from [128]. Copyright ©2020, Elsevier

A study showed that polydopamine-coated AuNSs-based hyperthermia-responsive nanoconstructs could significantly deliver pifithrin-μ, an inhibitor of HSPA5, for synergistic PTT (808 nm, 1 W/cm^2^, 5 min) and RT. These nanoconstructs showed hyperthermia-responsive release of pifithrin-μ resulting in the amplification of UPR in cancer cells (Fig. 4). Further, these nanoconstructs can also monitor cancer growth in response to the therapy (CT & MRI) [128].

AuNPs-based nanosystems that are responsive to the endogenous stimuli of tumor microenvironment are considered the most ideal drug carriers and are widely explored for targeted drug delivery. This is because the encapsulated drugs can be delivered at a specific time, site, and desired. The tumor microenvironment has low pH than normal tissues. Thus, AuNP-based nanoconstructs composed of materials responsive/sensitive to pH of the environment respond to pH change and may swell or collapse, resulting in drug release.

Shiyuan et al. developed pH-/laser-responsive size-tunable AuNCs modified by carboxymethyl chitosan and ICG as combined PTT/RT/PA/near-infrared fluorescence (NIRF) imaging agent. These AuNCs (initial size about 50 nm) were first accumulated into large aggregates of about 1000 nm under the acidic microenvironment at the tumor site for enhanced tumor retention. Further, these AuNCs were dispersed into ultra-small AuNPs (about 5 nm diameter) under PTT for enhanced penetration and RT effect [63]. Therefore, the use of stimuli-responsive size-tunable AuNPs is suggested for both enhanced circulation and tumor penetration ability.

A study showed the development of thiol-PEGylated AuNRs decorated with mercaptopropionylhydrazide for pH-responsive drug release. In addition, these nanoconstructs were conjugated with DOX, a chemotherapeutic drug, and 5-ALA, a PSs, for combined CTX, PTT (808 nm, 2.0 W/cm^2^, 5 min), and PDT (635 nm, 0.5 W/cm^2^, 5 min) for breast cancer. They found that cancer cells could efficiently internalize these nanoconstructs, suppressing tumor growth without systemic toxicity [184].

**Fig. 5:**
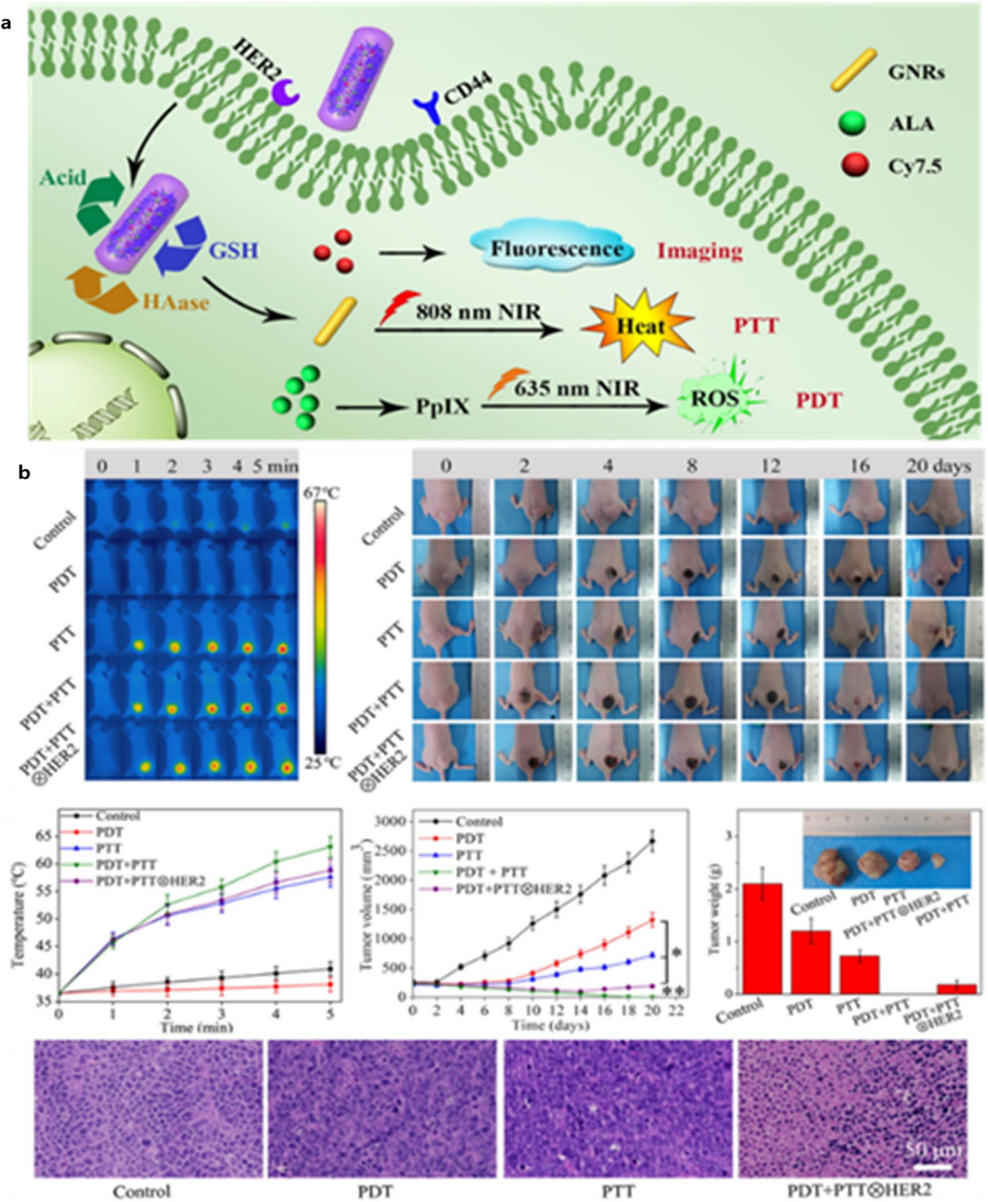
**a** Schematic representation, and **b** In vivo antitumor study in nude mice xenograft models of triple-responsive drug release from AuNR-HA^−ALA/Cy7.5^-HER2 for HER2/CD44 dual-targeted and fluorescence imaging-guided combined PDT/PTT treatment of breast cancer showing photothermal photographs, digital photos of mice bearing tumors, tumor temperature, tumor volume, tumor weight, and H&E stained micrographs of tumor tissues. Reproduced with permission from [186]. Copyright ©2019, Elsevier

An acid-triggered surface charge-reversal and pH/NIR dual-responsive aldehyde/catechol-functionalized hyaluronic acid and hydroxyethyl chitosan decorated AuNRs were developed for combined CTX/PTT for breast cancer at 750–900 nm and 2.0 W/cm^2^ for 5 min. These nanoconstructs were also efficiently internalized in MCF-7 breast cancer cells and reduced tumor growth [185]. In another study, AuNRs were decorated with 5-ALA, Cy7.5, and anti-HER2 antibody-conjugated hyaluronic acid to develop a dual-targeting and triple stimuli-responsive theranostic nanoplatform for both imaging and multi-modal therapeutics (Fig. 5) [186]. These nanoconstructs had a circulation half-life of 1.9 h, efficiently accumulated in the tumor tissue, and performed image-guided PDT/PTT treatments for breast cancer.

In addition, H_2_O_2_-responsive AuNCs were also studied for PDT and MRI. These AuNPs worked as an intelligent nanozyme for "off/on" modulation in response to oxygen. These AuNCs were loaded in mesoporous silica (mSiO_2_) and further wrapped in manganese dioxide (MnO_2_) nanosheets. It was found that in the presence of H_2_O_2_, the MnO_2_ shell degrades, switching "on" the PDT (under irradiation at 635 nm laser) and MRI activities. However, in normal physiological conditions, the MnO_2_ generates ^1^O_2_ that switches off MRI and PDT effects [187].

Thus, the AuNPs, in combination with stimuli-responsive polymers or molecules, can assist in the targeted delivery and stimuli-responsive release to maximize the therapeutic potential and minimize the undesired side-effect of Au-induced PTT and PDT effects.

### Current clinical approaches and key hurdles

Although various hyperthermia-mediated nanomedicines for cancer treatment are currently approved or in clinical trials [188], their clinical translation is still under exploration. Indeed, most hyperthermia-mediated nanomedicines utilize magnetic fields-mediated heat generation [189]. Among the biocompatible hyperthermia-mediated nanomedicines, AuNPs have been interested in inducing highly localized hyperthermia by converting the absorbed NIR light to heat, resulting in tumor ablation in preclinical animal models. Various AuNPs-based nanoconstructs are under clinical trials, as shown in Table 2.

**Table 2:** List of various AuNPs-based nanoconstructs under clinical trials

Interventions	Details	No. enrolled/age	Type of cancer	Phase	NCT number	Sponsor/collaborator	Summary
Aurimune (CYT-6091)	Tumor necrosis factor-bound colloidal AuNPs	60/18 years/adult, old adult	Advanced solid tumors	I	NCT00356980	National Institutes of Health Clinical Center	TNF-bound colloidal gold, may stimulate the immune system in different ways and stop tumor cells from growing
	Tumor necrosis factor-bound colloidal AuNPs	84/18 years and older	Breast, colon, gastrointestinal, kidney, liver, melanoma, ovarian, pancreatic, sarcoma, adrenocortical	I	NCT00436410	National Institutes of Health Clinical Center	This clinical trial is studying tumor necrosis factor in patients undergoing surgery for primary cancer or metastatic cancer
AureoLase^®^	Au nano-shells on silica core illumination with an 808 nm laser	11/18 years to 130 years	Head and neck cancer	Pilot study	NCT00848042	Nanospectra Biosciences, Inc	The tumor site is externally illuminated with NIR laser to activate the particles, resulting thermal ablation of the tumor
	Silica-gold nanoshells coated with PEG	1/18 years and older adult	Prostate	Pilot study	NCT01679470	Nanospectra Biosciences, Inc	Thermal ablation of tumor
	Au nanoshells	60/ 45 years and older adult	Prostate	Recruiting	NCT04240639	Nanospectra Biosciences, Inc	To determine the efficacy of using MRI/US fusion imaging technology to direct focal ablation of prostate tissue using nanoparticle-directed laser irradiation
		45/45 year and older adult	Prostate	Completed	NCT02680535	Nanospectra Biosciences, Inc	MRI/US Fusion imaging and biopsy in combination with nanoparticle directed Focal therapy for ablation of prostate tissue
NU-0129	Spherical Nucleic Acid (SNA) AuNPs	8/18 years and older	Glioblastoma	Phase 1	NCT03020017	Northwestern University/National Cancer Institute	Spherical Nucleic Acid AuNPs Targeting BCL2L12, NU-0129 can cross the blood brain barrier and stop cancer cells from growing

CYT-6091 is the first thiolated polyethylene glycol (PEG) coated and tumor necrosis factor-α conjugated AuNPs-based cancer therapy to reach early-phase clinical trials (NCT00436410) and phase I clinical trial (NCT00356980, NCT00436410). CYT-6091 combined with radiations showed significant breast tumor reduction in preclinical 4T1 breast carcinoma and SCC VII head and neck tumor squamous cell carcinoma mice models [190]. In phase I clinical trials, CYT-6091 showed no dose-limiting toxicity in clinical trials in a diverse set of advanced-stage cancer patients, including pancreatic ductal adenocarcinoma, breast cancer, and colon cancer, and the presence of AuNPs (CYT-6091) was observed in tumor tissue [191]. The National Cancer Institute (NCI) has planned for phase II clinical studies in 2021/2022 for CYT-6091 in combination with Abraxane (nab-paclitaxel) in patients with late-stage endocrine cancers of the pancreas and thyroid (CytImmune, https://www.cytimmune.com/pipelilne) (accessed on 7th March 2022).

AuroShells (AuNSs on silica, Aurolase^®^) is also approved in clinical trials for AuNPs-mediated PTT ablation of solid tumors via converting NIR light signals into heat. The Aurolase^®^ is ~ 150 nm in diameter and is specially designed to absorb maximum NIR light at 800 nm and convert it to heat. The first clinical study on AuroShells (ClinicalTrials.gov Identifier: NCT00848042), as reported in https://clinicaltrials.gov/, was performed by Nanospectra Biosciences, Inc. This interventional study was started in April 2008 and was completed in August 2014. They used different doses of AuroLase Therapy (i.e., 4.5, 7.5, and 7.5 mL/Kg of AuroShell particles combined with one or multiple doses of laser at irradiation at 808 nm and 3.4, 4.5, and 5 watts, respectively) on 11 patients with Head and Neck cancer. Each group received a single dose infusion of AuroShell (TM) particles followed by interstitial illuminations with an 808 nm laser, followed by monitoring for 6 months. It was found that the treatment was followed by few adverse side effects [192].

Another clinical study with Aurolase^®^ was started in October 2012 (ClinicalTrials.gov Identifier: NCT01679470). In this study, a single dose of AuNPs was administered in patients with primary and/or metastatic lung tumors (with airway obstruction). Then, the PTT effect was triggered via bronchoscopy using an optical fiber emitting NIR light (testing the irradiation of an escalating dose) [193, 194]. However, this study was not completed and was terminated in June 2014.

In 2016, Nanospectra Biosciences, Inc. started another clinical trial in forty-five patients with neoplastic prostate cancer (ClinicalTrials.gov Identifier: NCT02680535). The patients received a single intravenous infusion of AuroShell particles 12–36 h before MRI/US-guided laser irradiation using an FDA-cleared laser and an interstitial optical fiber. The patients were evaluated for laser tumor ablation and adverse events at three months (primary endpoint) and again at one year after laser treatment. Although the study was completed in 2020, results are not posted yet on https://clinicaltrials.gov/. Very recently, Nanospectra Biosciences, Inc. started recruitment of a clinical extension study (ClinicalTrials.gov Identifier: NCT04240639) of AuroLase Therapy in the focal ablation of prostate tissue via nanoparticle directed irradiation in low to intermediate-risk localized prostate cancer.

**Fig. 6:**
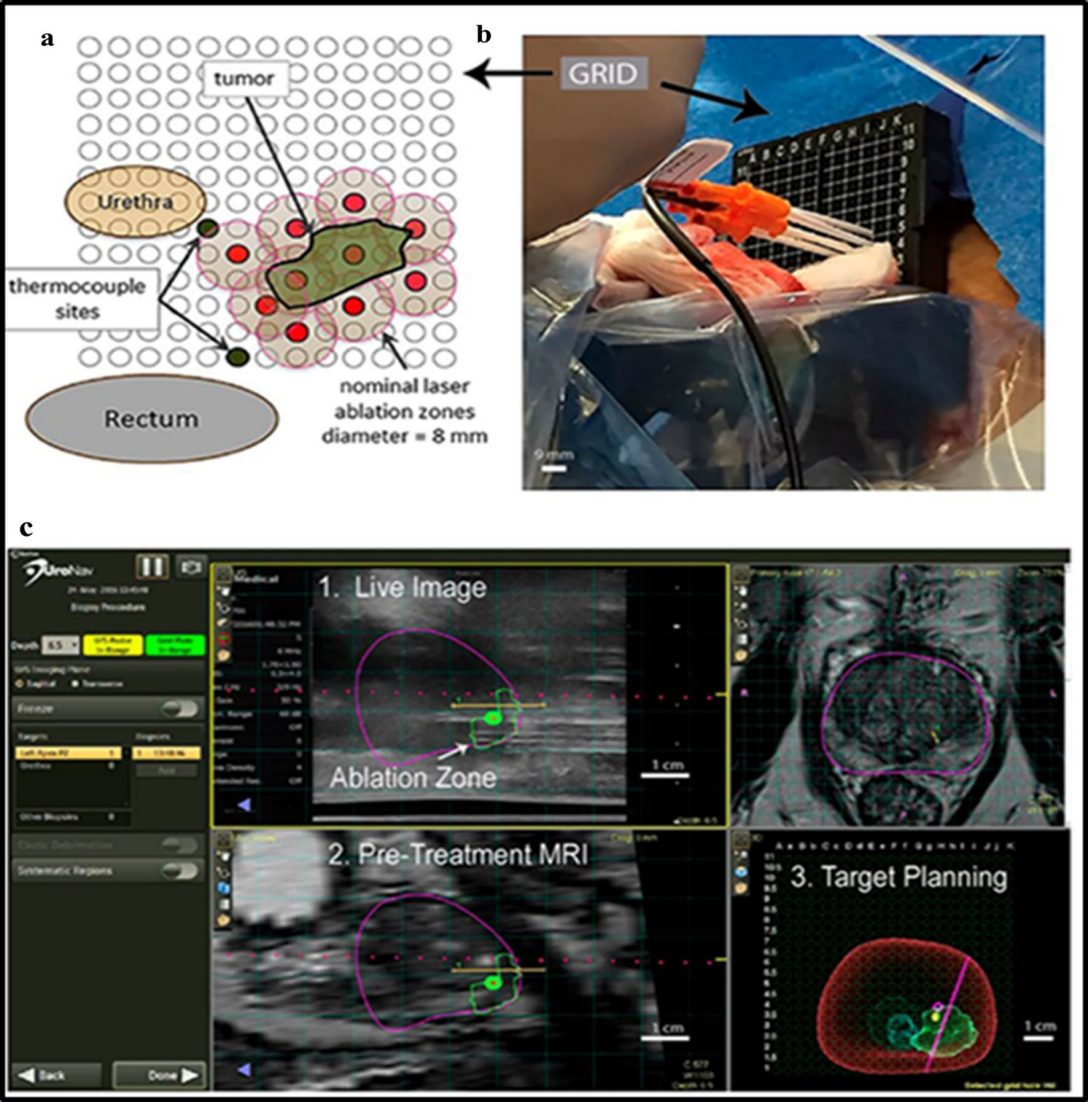
**a** Prostate ablation zone and the nearby urethra and rectum overlaid with a rectangular transperineal grid (3-mm spacing), **b** Laser introducers (orange hub) placed with the thermocouple (black) through the transperineal grid. **c** UroNav MR/US Fusion guidance for trocar placement with real-time ultrasound imaging. Reproduced with permission from [196]. Copyright ©2019, National Academy of Sciences

Although the results of the clinical trials on AuroLase therapy are pending, based on chemical, hematological, immunological, and urinalysis evaluations, it was found that the AuroShell particles have an excellent clinical safety profile in 22 patients with prostate cancer that matches the nonclinical findings [195]. Moreover, in a clinical pilot device study, Rastinehad et al. reported the feasibility and safety of laser-excited AuNSs treatment combined with MRI/US fusion imaging to treat low-intermediate-grade prostate tumors from 16 patients. The patients underwent AuroShell infusion and high-precision laser ablation, followed by a multiparametric prostate MRI at 48–72 h. After 3 and 12 months of the treatment, multiparametric high-resolution MRI/US targeted fusion biopsies and a standard 12-core systematic biopsy at 12 months were performed. It was found that 94% (15/16) of patients showed successful AuNSs-mediated focal laser ablation with no significant harmful changes in genitourinary function, indicating the feasibility and safety of AuroShell-directed laser excitation and ablation in men [196] (Fig. 6). Thus, suggesting that AuroShell nanoparticles can accumulate at the tumor site and can ablate prostate cancer with minimum side effects.

Moreover, before clinical translation, AuroLase^®^ still needs to face key hurdles, such as proving that their EPR effect can assist in their accumulation at the tumor site. Since the EPR effect is not well-proven in clinical trials, relying on only the EPR effect (not attachment with an active targeting agent) is challenging for AuroLase® [197]. Furthermore, since AuroLase^®^ is designated as local cancer therapy for solid tumors, treating systemic malignancies using AuroLase® is difficult and might need further modification [198].

Recently, a new term, “Nano-Ayurvedic Medicine,” was coined by Khoobchandani et al., recently approved by the US Patents and Trade Marks office [199]. They used AuNPs and a combination of phytochemicals to produce Nano Swarna Bhasma (NSB). This group first performed pre-clinical investigations on breast cancer-bearing mice and later moved to clinical trials in human patients. They found 100% clinical benefits in patients treated with NSB capsules, along with “standard of care treatment”. These results indicate that green nanotechnology presents promising opportunities for highly effective interventions to treat cancer patients.

Despite the tremendous efforts invested in developing AuNPs-based nanoconstructs for cancer treatment, more challenges and rooms still exist. As observed, very few clinical trials associated with AuNPs-based cancer therapy have been conducted till now. Thus, a lack of understandable information on the therapeutic effect and side effects of AuNPs-based cancer therapy can negatively influence human health. Therefore, more precise information on the long-term toxicity and chain reactions of AuNPs is required. For this purpose, clinically relevant organ-on-a-chip models and high-throughput assays can be used in addition to clinical toxicity assessments. Thus, the progress in clinical trials and the safety profile of AuNPs suggest their promising application to treat cancer. However, there is a need for more advanced research and collaborations of researchers from various sciences, such as biomedical, material science, and clinicians, to improvise the use of AuNPs for effective multimodal therapy against cancer.

## Conclusion

Compared to other traditional hyperthermia-mediated cancer treatments, AuNPs-mediated photothermal therapy (PTT) can target and ablate tumor cells because AuNPs can accumulate in the tumor microenvironment and tumor cells via extravasation tumor vascular network. Various in vitro and in vivo studies confirm the tumor ablation property of AuNPs in different tumor models. AuNPs-mediated PTT effect can be combined with other therapies, including photodynamic therapy, immunotherapy, radiotherapy, etc. Moreover, attaching the targeting agents on the surface of AuNPs increases the targeting ability of the AuNPs, resulting in increased hyperthermia-mediated cancer ablation. Along with primary tumors, these combinational therapies might also treat distant metastatic tumors. However, developing modest, effective, and feasible Au-based nanoconstructs for combinational therapies against cancer is still challenging and requires a collaborative effort from researchers from different streams.

## Data Availability

Not applicable.

## References

[1] Wen H, Tamarov K, Happonen E, Lehto V, Xu W (2020). Inorganic nanomaterials for photothermal-based cancer theranostics. Adv Therap.

[2] Bosset J-F, Collette L, Calais G, Mineur L, Maingon P, Radosevic-Jelic L (2006). Chemotherapy with preoperative radiotherapy in rectal cancer. N Engl J Med.

[3] Kim Y, Tomé WA (2006). Risk-adaptive optimization: selective boosting of high-risk tumor subvolumes. Int J Radiat Oncol Biol Phys.

[4] Jorfi M, Foster EJ (2015). Recent advances in nanocellulose for biomedical applications. J Appl Polym Sci.

[5] Yao C, Zhang L, Wang J, He Y, Xin J, Wang S (2016). Gold nanoparticle mediated phototherapy for cancer. J Nanomater.

[6] Ye F, Zhao Y, El-Sayed R, Muhammed M, Hassan M (2018). Advances in nanotechnology for cancer biomarkers. Nano Today.

[7] Alle M, Reddy B, Natarajan S (2014). Efficient pH dependent drug delivery to target cancer cells by gold nanoparticles capped with carboxymethyl chitosan. Int J Mol Sci.

[8] Yang G, Phua SZF, Bindra AK, Zhao Y (2019). Degradability and clearance of inorganic nanoparticles for biomedical applications. Adv Mater.

[9] Alle M, Park SC, Bandi R, Lee S-H, Kim J-C (2021). Rapid in-situ growth of gold nanoparticles on cationic cellulose nanofibrils: recyclable nanozyme for the colorimetric glucose detection. Carbohydr Polym.

[10] Fratoddi I, Venditti I, Cametti C, Russo MV (2015). How toxic are gold nanoparticles? The state-of-the-art. Nano Res.

[11] Her S, Jaffray DA, Allen C (2017). Gold nanoparticles for applications in cancer radiotherapy: mechanisms and recent advancements. Adv Drug Deliv Rev.

[12] Huang X, Kang B, Qian W, Mackey MA, Chen PC, Oyelere AK (2010). Comparative study of photothermolysis of cancer cells with nuclear-targeted or cytoplasm-targeted gold nanospheres: continuous wave or pulsed lasers. J Biomed Opt.

[13] Amina SJ, Guo B (2020). A review on the synthesis and functionalization of gold nanoparticles as a drug delivery vehicle. Int J Nanomedicine.

[14] Beik J, Khateri M, Khosravi Z, Kamrava SK, Kooranifar S, Ghaznavi H (2019). Gold nanoparticles in combinatorial cancer therapy strategies. Coord Chem Rev.

[15] Liu Y, Crawford BM, Vo-Dinh T (2018). Gold nanoparticles-mediated photothermal therapy and immunotherapy. Immunotherapy.

[16] Carnovale C, Bryant G, Shukla R, Bansal V (2019). Identifying trends in gold nanoparticle toxicity and uptake: size, shape, capping ligand, and biological corona. ACS Omega.

[17] Medici S, Peana M, Coradduzza D, Zoroddu MA (2021). Gold nanoparticles and cancer: detection, diagnosis and therapy. Semin Cancer Biol.

[18] Xie X, Liao J, Shao X, Li Q, Lin Y (2017). The effect of shape on cellular uptake of gold nanoparticles in the forms of stars, rods, and triangles. Sci Rep.

[19] Foroozandeh P, Aziz AA (2018). Insight into cellular uptake and intracellular trafficking of nanoparticles. Nanoscale Res Lett.

[20] Chen H, Kou X, Yang Z, Ni W, Wang J (2008). Shape- and size-dependent refractive index sensitivity of gold nanoparticles. Langmuir.

[21] Amendola V, Pilot R, Frasconi M, Maragò OM, Iatì MA (2017). Surface plasmon resonance in gold nanoparticles: a review. J Phys Condens Matter.

[22] Yuan F, Chen H, Xu J, Zhang Y, Wu Y, Wang L (2014). Aptamer-based luminescence energy transfer from near-infrared-to-near- infrared upconverting nanoparticles to gold nanorods and its application for the detection of thrombin. Chem A Eur J.

[23] Kim TH, Alle M, Park SC, Zhao F, Long W, Samala S (2021). Self-assembly prepared using an ion pair of poly(ethylene imine) and (phenylthio) acetic acid as a drug carrier for oxidation, temperature, and NIR-responsive release. Chem Eng J.

[24] Huang X, El-Sayed IH, Qian W, El-Sayed MA (2006). Cancer cell imaging and photothermal therapy in the near-infrared region by using gold nanorods. J Am Chem Soc.

[25] Dang X, Bardhan NM, Qi J, Gu L, Eze NA, Lin CW (2019). Deep-tissue optical imaging of near cellular-sized features. Sci Rep.

[26] Zhang A, Guo W, Qi Y, Wang J, Ma X, Yu D (2016). Synergistic effects of gold nanocages in hyperthermia and radiotherapy treatment. Nanoscale Res Lett.

[27] Hwang S, Nam J, Jung S, Song J, Doh H, Kim S (2014). Gold nanoparticle-mediated photothermal therapy: current status and future perspective. Nanomedicine.

[28] Nam J, Son S, Ochyl LJ, Kuai R, Schwendeman A, Moon JJ (2018). Chemo-photothermal therapy combination elicits anti-tumor immunity against advanced metastatic cancer. Nat Commun.

[29] Xia F, Hou W, Zhang C, Zhi X, Cheng J, de la Fuente JM (2018). pH-responsive gold nanoclusters-based nanoprobes for lung cancer targeted near-infrared fluorescence imaging and chemo-photodynamic therapy. Acta Biomater.

[30] Zhang C, Ren J, Hua J, Xia L, He J, Huo D (2018). Multifunctional Bi 2 WO 6 nanoparticles for CT-guided photothermal and oxygen-free photodynamic therapy. ACS Appl Mater Interfaces.

[31] Vijayaraghavan P, Liu CH, Vankayala R, Chiang CS, Hwang KC (2014). Designing multi-branched gold nanoechinus for NIR light activated dual modal photodynamic and photothermal therapy in the second biological window. Adv Mater.

[32] Sperling RA, Gil PR, Zhang F, Zanella M, Parak WJ (2008). Biological applications of gold nanoparticles. Chem Soc Rev.

[33] Sotiropoulos M, Henthorn NT, Warmenhoven JW, Mackay RI, Kirkby KJ, Merchant MJ (2017). Modelling direct DNA damage for gold nanoparticle enhanced proton therapy. Nanoscale.

[34] Kim M, Lee J, Nam J (2019). Plasmonic photothermal nanoparticles for biomedical applications. Adv Sci.

[35] Ahmad T, Sarwar R, Iqbal A, Bashir U, Farooq U, Halim SA (2020). Recent advances in combinatorial cancer therapy via multifunctionalized gold nanoparticles. Nanomedicine.

[36] Hasan W, Stender CL, Lee MH, Nehl CL, Lee J (2009). Tailoring the structure of nanopyramids for optimal heat generation. Nano Lett.

[37] Li Q, Zhuo X, Li S, Ruan Q, Xu QH, Wang J (2015). Production of monodisperse gold nanobipyramids with number percentages approaching 100% and evaluation of their plasmonic properties. Adv Opt Mater.

[38] Zhou G, Yang Y, Han S, Chen W, Fu Y, Zou C (2013). Growth of nanobipyramid by using large sized Au decahedra as seeds. ACS Appl Mater Interfaces.

[39] Liang S, Sun M, Lu Y, Shi S, Yang Y, Lin Y (2020). Cytokine-induced killer cells-assisted tumor-targeting delivery of Her-2 monoclonal antibody-conjugated gold nanostars with NIR photosensitizer for enhanced therapy of cancer. J Mater Chem B.

[40] Depciuch J, Stec M, Maximenko A, Pawlyta M, Baran J, Parlinska-Wojtan M (2019). Control of arms of Au stars size and its dependent cytotoxicity and photosensitizer effects in photothermal anticancer therapy. Int J Mol Sci.

[41] Hu M, Chen J, Li ZY, Au L, Hartland GV, Li X (2006). Gold nanostructures: engineering their plasmonic properties for biomedical applications. Chem Soc Rev.

[42] Ma X, Cheng Y, Huang Y, Tian Y, Wang S, Chen Y (2015). PEGylated gold nanoprisms for photothermal therapy at low laser power density. RSC Adv.

[43] Alfranca G, Artiga Á, Stepien G, Moros M, Mitchell SG, De La Fuente JM (2016). Gold nanoprism–nanorod face off: comparing the heating efficiency, cellular internalization and thermoablation capacity. Nanomedicine.

[44] Pelaz B, Grazu V, Ibarra A, Magen C, Del Pino P, De La Fuente JM (2012). Tailoring the synthesis and heating ability of gold nanoprisms for bioapplications. Langmuir.

[45] Alfranca G, Beola L, Liu Y, Gutiérrez L, Zhang A, Artiga A (2019). In vivo comparison of the biodistribution and long-term fate of colloids—gold nanoprisms and nanorods—with minimum surface modification. Nanomedicine.

[46] Yim W, Borum RM, Zhou J, Mantri Y, Wu Z, Zhou J (2022). Ultrasmall gold nanorod-polydopamine hybrids for enhanced photoacoustic imaging and photothermal therapy in second near-infrared window. Nanotheranostics.

[47] Agabeigi R, Rasta SH, Rahmati-Yamchi M, Salehi R, Alizadeh E (2020). Novel chemo-photothermal therapy in breast cancer using methotrexate-loaded folic acid conjugated Au@SiO2 nanoparticles. Nanoscale Res Lett.

[48] Tan T, Wang H, Cao H, Zeng L, Wang Y, Wang Z (2018). Deep tumor-penetrated nanocages improve accessibility to cancer stem cells for photothermal-chemotherapy of breast cancer metastasis. Adv Sci.

[49] Song J, Pu L, Zhou J, Duan B, Duan H (2013). Biodegradable theranostic plasmonic vesicles of amphiphilic gold nanorods. ACS Nano.

[50] Hone DC, Walker PI, Evans-Gowing R, FitzGerald S, Beeby A, Chambrier I (2002). Generation of cytotoxic singlet oxygen via phthalocyaninel-stabilized gold nanoparticles: a potential delivery vehicle for photodynamic therapy. Langmuir.

[51] Castilho ML, Jesus VPS, Vieira PFA, Hewitt KC, Raniero L (2021). Chlorin e6-EGF conjugated gold nanoparticles as a nanomedicine based therapeutic agent for triple negative breast cancer. Photodiagnosis Photodyn Ther.

[52] García Calavia P, Marín MJ, Chambrier I, Cook MJ, Russell DA (2018). Towards optimisation of surface enhanced photodynamic therapy of breast cancer cells using gold nanoparticle–photosensitiser conjugates. Photochem Photobiol Sci.

[53] Crous A, Abrahamse H. Effective gold nanoparticle-antibody-mediated drug delivery for photodynamic therapy of lung cancer Stem cells. Int J Mol Sci. Multidisciplinary Digital Publishing Institute (MDPI). 2020. https://www.ncbi.nlm.nih.gov/pmc/articles/PMC7311980/. Accessed 7 Dec 2021. 10.3390/ijms21113742 PMC7311980 32466428

[54] Wang J, Zhuo X, Xiao X, Mao R, Wang Y, Wang J (2019). AlPcS-loaded gold nanobipyramids with high two-photon efficiency for photodynamic therapy in vivo. Nanoscale.

[55] Li H, Wang P, Deng Y, Zeng M, Tang Y, Zhu WH (2017). Combination of active targeting, enzyme-triggered release and fluorescent dye into gold nanoclusters for endomicroscopy-guided photothermal/photodynamic therapy to pancreatic ductal adenocarcinoma. Biomaterials.

[56] Li B, Sun L, Li T, Zhang Y, Niu X, Xie M (2021). Ultra-small gold nanoparticles self-assembled by gadolinium ions for enhanced photothermal/photodynamic liver cancer therapy. J Mater Chem B.

[57] Depciuch J, Stec M, Klebowski B, Baran J, Parlinska-Wojtan M (2019). Platinum-gold nanoraspberries as effective photosensitizer in anticancer photothermal therapy. J Nanobiotechnol.

[58] Liu C, Luo L, Zeng L, Xing J, Xia Y, Sun S (2018). Porous Gold nanoshells on functional NH 2-MOFs: facile synthesis and designable platforms for cancer multiple therapy. Small.

[59] Chen Y, Yang J, Fu S, Wu J (2020). Gold nanoparticles as radiosensitizers in cancer radiotherapy. Int J Nanomedicine.

[60] Le Goas M, Paquet M, Paquirissamy A, Guglielmi J, Compin C, Thariat J (2019). Improving 131 I radioiodine therapy by hybrid polymer-grafted gold nanoparticles. Int J Nanomedicine.

[61] Zhang Y, Huang F, Ren C, Liu J, Yang L, Chen S (2019). Enhanced radiosensitization by gold nanoparticles with acid-triggered aggregation in cancer radiotherapy. Adv Sci.

[62] Xie J, Gong L, Zhu S, Yong Y, Gu Z, Zhao Y (2019). Emerging strategies of nanomaterial-mediated tumor radiosensitization. Adv Mater.

[63] Hua S, He J, Zhang F, Yu J, Zhang W, Gao L (2021). Multistage-responsive clustered nanosystem to improve tumor accumulation and penetration for photothermal/enhanced radiation synergistic therapy. Biomaterials.

[64] Zhang X, Chen X, Jiang Y-W, Ma N, Xia L-Y, Cheng X (2018). Glutathione-depleting gold nanoclusters for enhanced cancer radiotherapy through synergistic external and internal regulations. ACS Appl Mater Interfaces.

[65] Hetz C (2012). The unfolded protein response: controlling cell fate decisions under ER stress and beyond. Nat Rev Mol Cell Biol.

[66] Zhang F, Han X, Hu Y, Wang S, Liu S, Pan X (2019). Interventional photothermal therapy enhanced brachytherapy: a new strategy to fight deep pancreatic cancer. Adv Sci.

[67] Piccolo O, Lincoln JD, Melong N, Orr BC, Fernandez NR, Borsavage J (2022). Radiation dose enhancement using gold nanoparticles with a diamond linear accelerator target: a multiple cell type analysis. Sci Rep.

[68] Li D, Zhao J, Ma J, Yang H, Zhang X, Cao Y (2022). GMT8 aptamer conjugated PEGylated Ag@Au core-shell nanoparticles as a novel radiosensitizer for targeted radiotherapy of glioma. Colloids Surf B Biointerfaces.

[69] Mehrnia SS, Hashemi B, Mowla SJ, Nikkhah M, Arbabi A (2021). Radiosensitization of breast cancer cells using AS1411 aptamer-conjugated gold nanoparticles. Radiat Oncol.

[70] Hua Y, Wang Y, Kang X, Xu F, Han Z, Zhang C (2021). A multifunctional AIE gold cluster-based theranostic system: tumor-targeted imaging and Fenton reaction-assisted enhanced radiotherapy. J Nanobiotechnol.

[71] He C, Zhang Z, Ding Y, Xue K, Wang X, Yang R (2021). LRP1-mediated pH-sensitive polymersomes facilitate combination therapy of glioblastoma in vitro and in vivo. J Nanobiotechnol.

[72] Luo D, Johnson A, Wang X, Li H, Erokwu BO, Springer S (2020). Targeted radiosensitizers for MR-guided radiation therapy of prostate cancer. Nano Lett.

[73] Ding Y, Sun Z, Tong Z, Zhang S, Min J, Xu Q (2020). Tumor microenvironment-responsive multifunctional peptide coated ultrasmall gold nanoparticles and their application in cancer radiotherapy. Theranostics.

[74] Bhattarai S, Mackeyev Y, Venkatesulu BP, Krishnan S, Singh PK (2021). CXC chemokine receptor 4 (CXCR4) targeted gold nanoparticles potently enhance radiotherapy outcomes in breast cancer. Nanoscale.

[75] Banstola A, Poudel K, Emami F, Ku SK, Jeong J-H, Kim JO (2021). Localized therapy using anti-PD-L1 anchored and NIR-responsive hollow gold nanoshell (HGNS) loaded with doxorubicin (DOX) for the treatment of locally advanced melanoma. Nanomedicine.

[76] Tian J, Gu Y, Li Y, Liu T (2020). CD271 antibody-functionalized HGNs for targeted photothermal therapy of osteosarcoma stem cells. Nanotechnology.

[77] Yook S, Cai Z, Jeong JJ, Lu Y, Winnik MA, Pignol J-P (2020). Dual-receptor-targeted (DRT) radiation nanomedicine labeled with 177Lu Is more potent for killing human breast cancer cells that coexpress HER2 and EGFR than single-receptor-targeted (SRT) radiation nanomedicines. Mol Pharm.

[78] Mantso T, Vasileiadis S, Anestopoulos I, Voulgaridou GP, Lampri E, Botaitis S (2018). Hyperthermia induces therapeutic effectiveness and potentiates adjuvant therapy with non-targeted and targeted drugs in an in vitro model of human malignant melanoma. Sci Rep.

[79] Liu Y, Chongsathidkiet P, Crawford BM, Odion R, Dechant CA, Kemeny HR (2019). Plasmonic gold nanostar-mediated photothermal immunotherapy for brain tumor ablation and immunologic memory. Immunotherapy.

[80] Madsen SJ, Christie C, Hong SJ, Trinidad A, Peng Q, Uzal FA (2015). Nanoparticle-loaded macrophage-mediated photothermal therapy: potential for glioma treatment. Lasers Med Sci.

[81] Rao L, Bu LL, Ma L, Wang W, Liu H, Wan D (2018). Platelet-facilitated photothermal therapy of head and neck squamous cell carcinoma. Angew Chem Int Ed Engl.

[82] Mooney R, Roma L, Zhao D, Van Haute D, Garcia E, Kim SU (2014). Neural stem cell-mediated intratumoral delivery of gold nanorods improves photothermal therapy. ACS Nano.

[83] Liu Y, Yang M, Zhang J, Zhi X, Li C, Zhang C (2016). Human induced pluripotent stem cells for tumor targeted delivery of gold nanorods and enhanced photothermal therapy. ACS Nano.

[84] Wu J, Liu Y, Tang Y, Wang S, Wang C, Li Y (2016). Synergistic chemo-photothermal therapy of breast cancer by mesenchymal stem cell-encapsulated yolk-shell GNR@HPMO-PTX nanospheres. ACS Appl Mater.

[85] Huang L, Xu C, Xu P, Qin Y, Chen M, Feng Q (2019). Intelligent photosensitive mesenchymal stem cells and cell-derived microvesicles for photothermal therapy of prostate cancer. Nanotheranostics.

[86] Choi J, Kim HY, Ju EJ, Jung J, Park J, Chung HK (2012). Use of macrophages to deliver therapeutic and imaging contrast agents to tumors. Biomaterials.

[87] Huang B, Abraham WD, Zheng Y, Bustamante López SC, Luo SS, Irvine DJ. Active targeting of chemotherapy to disseminated tumors using nanoparticle-carrying T cells. Sci Transl Med. 2015. https://pubmed.ncbi.nlm.nih.gov/26062846/. Accessed 7 Dec 2021. 10.1126/scitranslmed.aaa5447 PMC4687972 26062846

[88] Huang WC, Chiang WH, Cheng YH, Lin WC, Yu CF, Yen CY (2015). Tumortropic monocyte-mediated delivery of echogenic polymer bubbles and therapeutic vesicles for chemotherapy of tumor hypoxia. Biomaterials.

[89] Xue J, Zhao Z, Zhang L, Xue L, Shen S, Wen Y (2017). Neutrophil-mediated anticancer drug delivery for suppression of postoperative malignant glioma recurrence. Nat Nanotechnol.

[90] Stephan MT, Moon JJ, Um SH, Bersthteyn A, Irvine DJ (2010). Therapeutic cell engineering with surface-conjugated synthetic nanoparticles. Nat Med.

[91] Gournaris E, Park W, Cho S, Bentrem DJ, Larson AC, Kim DH (2019). Near-infrared fluorescent endoscopic image-guided photothermal ablation therapy of colorectal cancer using dual-modal gold nanorods targeting tumor-infiltrating innate immune cells in a transgenic TS4 CRE/APCloxÎ"468 mouse model. ACS Appl Mater Interfaces.

[92] Ye B, Zhao B, Wang K, Guo Y, Lu Q, Zheng L (2020). Neutrophils mediated multistage nanoparticle delivery for prompting tumor photothermal therapy. J Nanobiotechnol.

[93] Liu B, Cao W, Cheng J, Fan S, Pan S, Wang L (2019). Human natural killer cells for targeting delivery of gold nanostars and bimodal imaging directed photothermal/photodynamic therapy and immunotherapy. Cancer Biol Med.

[94] Brenner JS, Mitragotri S, Muzykantov VR (2021). Red blood cell hitchhiking: a novel approach for vascular delivery of nanocarriers. Annu Rev Biomed Eng.

[95] Zhu H, Li Y, Ming Z, Liu W (2021). Glucose oxidase-mediated tumor starvation therapy combined with photothermal therapy for colon cancer. Biomater Sci.

[96] Singh P, Pandit S, Mokkapati VRSS, Garg A, Ravikumar V, Mijakovic I (2018). Gold nanoparticles in diagnostics and therapeutics for human cancer. Int J Mol Sci.

[97] Luo D, Wang X, Burda C, Basilion JP (2021). Recent development of gold nanoparticles as contrast agents for cancer diagnosis. Cancers.

[98] Mahato K, Nagpal S, Shah MA, Srivastava A, Maurya PK, Roy S (2019). Gold nanoparticle surface engineering strategies and their applications in biomedicine and diagnostics. 3 Biotech.

[99] Zou L, Wang H, He B, Zeng L, Tan T, Cao H (2016). Current approaches of photothermal therapy in treating cancer metastasis with nanotherapeutics. Theranostics.

[100] Cheng X, Sun R, Yin L, Chai Z, Shi H, Gao M (2017). Light-triggered assembly of gold nanoparticles for photothermal therapy and photoacoustic imaging of tumors in vivo. Adv Mater.

[101] Li W, Chen X (2015). Gold nanoparticles for photoacoustic imaging. Nanomedicine.

[102] Blasiak B, van Veggel FCJM, Tomanek B (2013). Applications of nanoparticles for MRI cancer diagnosis and therapy. J Nanomater.

[103] Khan M, Boumati S, Arib C, Diallo AT, Djaker N, Doan BT (2021). Doxorubicin (DOX) gadolinium–gold-complex: a new way to tune hybrid nanorods as theranostic agent. Int J Nanomedicine.

[104] Mahan MM, Doiron AL (2018). Gold nanoparticles as X-Ray, CT, and multimodal imaging contrast agents: formulation, targeting, and methodology. J Nanomater.

[105] Dong YC, Hajfathalian M, Maidment PSN, Hsu JC, Naha PC, Si-Mohamed S (2019). Effect of gold nanoparticle size on their properties as contrast agents for computed tomography. Sci Rep.

[106] Liu R, Guo H, Ouyang Z, Fan Y, Cao X, Xia J (2021). Multifunctional core-shell tecto dendrimers incorporated with gold nanoparticles for targeted dual mode CT/MR imaging of tumors. ACS Appl bio Mater.

[107] Li Q, Tang Q, Zhang P, Wang Z, Zhao T, Zhou J (2015). Human epidermal growth factor receptor-2 antibodies enhance the specificity and anticancer activity of light-sensitive doxorubicin-labeled liposomes. Biomaterials.

[108] Iqbal N, Iqbal N (2014). Human epidermal growth factor receptor 2 (HER2) in cancers: overexpression and therapeutic implications. Mol Biol Int.

[109] Dong Q, Yang H, Wan C, Zheng D, Zhou Z, Xie S (2019). Her2-functionalized gold-nanoshelled magnetic hybrid nanoparticles: a theranostic agent for dual-modal imaging and photothermal therapy of breast cancer. Nanoscale Res Lett.

[110] Kubota T, Kuroda S, Kanaya N, Morihiro T, Aoyama K, Kakiuchi Y (2018). HER2-targeted gold nanoparticles potentially overcome resistance to trastuzumab in gastric cancer. Nanomedicine.

[111] Elzoghby AO, Samy WM, Elgindy NA (2012). Albumin-based nanoparticles as potential controlled release drug delivery systems. J Control Release.

[112] Mocan L, Matea C, Tabaran FA, Mosteanu O, Pop T, Mocan T (2016). Photothermal treatment of liver cancer with albumin-conjugated gold nanoparticles initiates Golgi apparatus–ER dysfunction and caspase-3 apoptotic pathway activation by selective targeting of Gp60 receptor. Int J Nanomedicine.

[113] Llovet JM, Kelley RK, Villanueva A, Singal AG, Pikarsky E, Roayaie S (2021). Hepatocellular carcinoma. Nat Rev Dis Prim.

[114] Blachier M, Leleu H, Peck-Radosavljevic M, Valla DC, Roudot-Thoraval F (2013). The burden of liver disease in Europe: a review of available epidemiological data. J Hepatol.

[115] Park S, Kim H, Lim SC, Lim K, Lee ES, Oh KT (2019). Gold nanocluster-loaded hybrid albumin nanoparticles with fluorescence-based optical visualization and photothermal conversion for tumor detection/ablation. J Control Release.

[116] Xia F, Niu J, Hong Y, Li C, Cao W, Wang L (2019). Matrix metallopeptidase 2 targeted delivery of gold nanostars decorated with IR-780 iodide for dual-modal imaging and enhanced photothermal/photodynamic therapy. Acta Biomater.

[117] Zhang Y, Zhou L, Tan J, Liu J, Shan X, Ma Y (2020). Laser-triggered collaborative chemophotothermal effect of gold nanoparticles for targeted colon cancer therapy. Biomed Pharmacother.

[118] Mulens-Arias V, Nicolás-Boluda A, Pinto A, Balfourier A, Carn F, Silva AKA (2021). Tumor-selective immune-active mild hyperthermia associated with chemotherapy in colon peritoneal metastasis by photoactivation of fluorouracil-gold nanoparticle complexes. ACS Nano.

[119] Yu M, Zhou C, Liu J, Hankins JD, Zheng J (2011). Luminescent gold nanoparticles with pH-dependent membrane adsorption. J Am Chem Soc.

[120] Liu J, Duchesne PN, Yu M, Jiang X, Ning X, Vinluan RD (2016). Luminescent gold nanoparticles with size-independent emission. Angew Chem.

[121] Steckiewicz KP, Barcinska E, Sobczak K, Tomczyk E, Wojcik M, Inkielewicz-Stepniak I (2020). Assessment of anti-tumor potential and safety of application of glutathione stabilized gold nanoparticles conjugated with chemotherapeutics. Int J Med Sci.

[122] Buonerba A, Lapenta R, Donniacuo A, Licasale M, Vezzoli E, Milione S (2020). NIR multiphoton ablation of cancer cells, fluorescence quenching and cellular uptake of dansyl-glutathione-coated gold nanoparticles. Sci Rep.

[123] Vinluan RD, Liu J, Zhou C, Yu M, Yang S, Kumar A (2014). Glutathione-coated luminescent gold nanoparticles: a surface ligand for minimizing serum protein adsorption. ACS Appl Mater Interfaces.

[124] Yang H, He H, Tong Z, Xia H, Mao Z, Gao C (2020). The impact of size and surface ligand of gold nanorods on liver cancer accumulation and photothermal therapy in the second near-infrared window. J Colloid Interface Sci.

[125] Chebil C, Boumediene F, Cicero CE, Rascunà C, Di Prima A, Maria Torrisi AA (2021). Incidence, survival and geoepidemiological analysis of meningiomas and glioblastomas in the province of Catania during the 2003–2016 period. Environ Res.

[126] Cramer SW, Chen CC (2019). Photodynamic therapy for the treatment of glioblastoma. Front Surg.

[127] Ziu M, Goyal S, Olson JJ (2021). Congress of neurological surgeons systematic review and evidence-based guidelines update on the role of radiation therapy in the management of progressive and recurrent glioblastoma in adults. J Neurooncol.

[128] Zhu H, Cao X, Cai X, Tian Y, Wang D, Qi J, et al. Pifithrin-μ incorporated in gold nanoparticle amplifies pro-apoptotic unfolded protein response cascades to potentiate synergistic glioblastoma therapy. Biomaterials. 2020;232. https://pubmed.ncbi.nlm.nih.gov/31865193/. Accessed 7 Dec 2021. 10.1016/j.biomaterials.2019.119677 31865193

[129] Kim HS, Lee SJ, Lee DY (2022). Milk protein-shelled gold nanoparticles with gastrointestinally active absorption for aurotherapy to brain tumor. Bioact Mater.

[130] Joseph C, Daniels A, Singh S, Singh M (2021). Histidine-tagged folate-targeted gold nanoparticles for enhanced transgene expression in breast cancer cells in vitro. Pharmaceutics.

[131] Keyvan Rad J, Mahdavian AR, Khoei S, Shirvalilou S (2018). Enhanced photogeneration of reactive oxygen species and targeted photothermal therapy of C6 glioma brain cancer cells by folate-conjugated gold-photoactive polymer nanoparticles. ACS Appl Mater Interfaces.

[132] Kumar SSD, Mahesh A, Antoniraj MG, Rathore HS, Houreld NN, Kandasamy R (2018). Cellular imaging and folate receptor targeting delivery of gum kondagogu capped gold nanoparticles in cancer cells. Int J Biol Macromol.

[133] Mirrahimi M, Hosseini V, Kamrava SK, Attaran N, Beik J, Kooranifar S (2018). Selective heat generation in cancer cells using a combination of 808 nm laser irradiation and the folate-conjugated Fe2O3@Au nanocomplex. Artif Cells Nanomed Biotechnol.

[134] Yi M, Niu M, Xu L, Luo S, Wu K (2021). Regulation of PD-L1 expression in the tumor microenvironment. J Hematol Oncol.

[135] Han Y, Liu D, Li L (2020). PD-1/PD-L1 pathway: current researches in cancer. Am J Cancer Res.

[136] Gonzalez-Cao M, Morán T, Dalmau J, Garcia-Corbacho J, Bracht JWP, Bernabe R (2020). Assessment of the feasibility and safety of durvalumab for treatment of solid tumors in patients with HIV-1 infection: the phase 2 DURVAST study. JAMA Oncol.

[137] Choi BBR, Choi J-H, Kim UK, Hwang DS, Kim GC (2021). Gold nanoparticles conjugated with programmed death-ligand 1 antibodies induce apoptosis of SCC-25 oral squamous cell carcinoma cells via programmed death-ligand 1/signal transducer and transcription 3 pathway. Arch Oral Biol.

[138] Liu B, Qiao G, Han Y, Shen E, Alfranca G, Tan H (2020). Targeted theranostics of lung cancer: PD-L1-guided delivery of gold nanoprisms with chlorin e6 for enhanced imaging and photothermal/photodynamic therapy. Acta Biomater.

[139] Maiuthed A, Chantarawong W, Chanvorachote P (2018). Lung cancer stem cells and cancer stem cell-targeting natural compounds. Anticancer Res.

[140] Phi LTH, Sari IN, Yang YG, Lee SH, Jun N, Kim KS, et al. Cancer Stem Cells (CSCs) in drug resistance and their therapeutic implications in cancer treatment. Stem Cells Int. 2018. https://pubmed.ncbi.nlm.nih.gov/29681949/. Accessed 7 Dec 2021. 10.1155/2018/5416923 PMC5850899 29681949

[141] Emami F, Banstola A, Vatanara A, Lee S, Kim JO, Jeong J-H (2019). Doxorubicin and anti-PD-L1 antibody conjugated gold nanoparticles for colorectal cancer photochemotherapy. Mol Pharm.

[142] Nieberler M, Reuning U, Reichart F, Notni J, Wester H-J, Schwaiger M (2017). Exploring the role of RGD-recognizing integrins in cancer. Cancers.

[143] Garrigues HJ, Rubinchikova YE, Dipersio CM, Rose TM (2008). Integrin alphaVbeta3 Binds to the RGD motif of glycoprotein B of Kaposi’s sarcoma-associated herpesvirus and functions as an RGD-dependent entry receptor. J Virol.

[144] Wu P-H, Onodera Y, Ichikawa Y, Rankin EB, Giaccia AJ, Watanabe Y (2017). Targeting integrins with RGD-conjugated gold nanoparticles in radiotherapy decreases the invasive activity of breast cancer cells. Int J Nanomedicine.

[145] Song C, Li F, Guo X, Chen W, Dong C, Zhang J (2019). Gold nanostars for cancer cell-targeted SERS-imaging and NIR light-triggered plasmonic photothermal therapy (PPTT) in the first and second biological windows. J Mater Chem B.

[146] Albertini B, Mathieu V, Iraci N, Van Woensel M, Schoubben A, Donnadio A (2019). Tumor targeting by peptide-decorated gold nanoparticles. Mol Pharm.

[147] Faintuch BL, Teodoro R, Duatti A, Muramoto E, Faintuch S, Smith CJ (2008). Radiolabeled bombesin analogs for prostate cancer diagnosis: preclinical studies. Nucl Med Biol.

[148] Xu H, Sheng G, Lu L, Wang C, Zhang Y, Feng L (2021). GRPr-mediated photothermal and thermodynamic dual-therapy for prostate cancer with synergistic anti-apoptosis mechanism. Nanoscale.

[149] Lowrance WT, Murad MH, Oh WK, Jarrard DF, Resnick MJ, Cookson MS (2018). Castration-resistant prostate cancer: AUA guideline amendment 2018. J Urol.

[150] Ito K (2014). Prostate cancer in Asian men. Nat Rev Urol.

[151] Wong YNS, Ferraldeschi R, Attard G, De Bono J (2014). Evolution of androgen receptor targeted therapy for advanced prostate cancer. Nat Rev Clin Oncol.

[152] Gu C, Li C, Zhang J, Li X, Wang L, Ju Y, et al. Ultra-effective near-infrared Photothermal therapy for the prostate cancer Nursing care through novel intended and surface tailored photo-responsive Ga-Au@MPS nanovesicles. J Photochem Photobiol B. 2020 https://pubmed.ncbi.nlm.nih.gov/31810035/. Accessed 8 Dec 2021. 10.1016/j.jphotobiol.2019.111685 31810035

[153] Cookson MS, Roth BJ, Dahm P, Engstrom C, Freedland SJ, Hussain M (2013). Castration-resistant prostate cancer: AUA guideline. J Urol.

[154] Nuhn P, De Bono JS, Fizazi K, Freedland SJ, Grilli M, Kantoff PW (2019). Update on systemic prostate cancer therapies: management of metastatic castration-resistant prostate cancer in the era of precision oncology. Eur Urol.

[155] Tan H, Hou N, Liu Y, Liu B, Cao W, Zheng D, et al. CD133 antibody targeted delivery of gold nanostars loading IR820 and docetaxel for multimodal imaging and near-infrared photodynamic/photothermal/chemotherapy against castration resistant prostate cancer. Nanomedicine. 2020;27. https://pubmed.ncbi.nlm.nih.gov/32229215/. Accessed 8 Dec 2021. 10.1016/j.nano.2020.102192 32229215

[156] Carrera S, Sancho A, Azkona E, Azkuna J, Lopez-Vivanco G (2017). Hereditary pancreatic cancer: related syndromes and clinical perspective. Hered Cancer Clin Pract.

[157] Westphalen CB, Kruger S, Haas M, Heinemann V, Boeck S (2016). Safety of palliative chemotherapy in advanced pancreatic cancer. Expert Opin Drug Saf.

[158] Thapa RK, Choi Y, Jeong JH, Youn YS, Choi HG, Yong CS (2016). Folate-mediated targeted delivery of combination chemotherapeutics loaded reduced graphene oxide for synergistic chemo-photothermal therapy of cancers. Pharm Res.

[159] Joubert F, Pasparakis G (2018). Hierarchically designed hybrid nanoparticles for combinational photochemotherapy against a pancreatic cancer cell line. J Mater Chem B.

[160] Emamzadeh M, Pasparakis G (2021). Polymer coated gold nanoshells for combinational photochemotherapy of pancreatic cancer with gemcitabine. Sci Rep.

[161] Banstola A, Pham TT, Jeong JH, Yook S (2019). Polydopamine-tailored paclitaxel-loaded polymeric microspheres with adhered NIR-controllable gold nanoparticles for chemo-phototherapy of pancreatic cancer. Drug Deliv.

[162] Jo H, Youn H, Lee S, Ban C (2014). Ultra-effective photothermal therapy for prostate cancer cells using dual aptamer-modified gold nanostars. J Mater Chem B.

[163] Mangadlao JD, Wang X, McCleese C, Escamilla M, Ramamurthy G, Wang Z (2018). Prostate-specific membrane antigen targeted gold nanoparticles for theranostics of prostate cancer. ACS Nano.

[164] Adil MS, Khulood D, Somanath PR (2021). Targeting Akt-associated microRNAs for cancer therapeutics. Biochem Pharmacol.

[165] Khar R, Warsi M, Akhter S, Ahmad F, Jain G, Mallick N (2010). Nano-vectors for the ocular delivery of nucleic acid-based therapeutics. Indian J Pharm Sci.

[166] Ferreira D, Fontinha D, Martins C, Pires D, Fernandes AR, Baptista PV (2020). Gold nanoparticles for vectorization of nucleic acids for cancer therapeutics. Molecules.

[167] Luther DC, Huang R, Jeon T, Zhang X, Lee Y-W, Nagaraj H (2020). Delivery of drugs, proteins, and nucleic acids using inorganic nanoparticles. Adv Drug Deliv Rev.

[168] Liu B, Cao W, Qiao G, Yao S, Pan S, Wang L (2019). Effects of gold nanoprism-assisted human PD-L1 siRNA on both gene down-regulation and photothermal therapy on lung cancer. Acta Biomater.

[169] Peng J, Wang R, Sun W, Huang M, Wang R, Li Y (2021). Delivery of miR-320a-3p by gold nanoparticles combined with photothermal therapy for directly targeting Sp1 in lung cancer. Biomater Sci.

[170] Abashkin V, Pędziwiatr-Werbicka E, Gómez R, de la Mata FJ, Dzmitruk V, Shcharbin D (2021). Prospects of cationic carbosilane dendronized gold nanoparticles as non-viral vectors for delivery of anticancer siRNAs siBCL-xL and siMCL-1. Pharmaceutics.

[171] Li J, Shen M, Shi X (2020). Poly(amidoamine) dendrimer-gold nanohybrids in cancer gene therapy: a concise overview. ACS Appl bio Mater.

[172] Zhuang M, Jiang S, Gu A, Chen X, Mingyan E (2021). Radiosensitizing effect of gold nanoparticle loaded with small interfering RNA-SP1 on lung cancer: AuNPs-si-SP1 regulates GZMB for radiosensitivity. Transl Oncol.

[173] Xu G, Zhang H, Li Z, Wu S, Quan R, Mao K, Sheng Y, Li X (2020). Effect of HIF-1αsiRNA-linked AuNRs on radiotherapy of nasopharyngeal carcinoma. Cell Mol Biol.

[174] Shrestha B, Wang L, Zhang H, Hung CY, Tang L (2020). Gold nanoparticles mediated drug-gene combinational therapy for breast cancer treatment. Int J Nanomedicine.

[175] Yue R, Chen M, Ma N (2020). Dual MicroRNA-triggered drug release system for combined chemotherapy and gene therapy with logic operation. ACS Appl Mater Interfaces.

[176] Xue C, Hu S, Gao Z-H, Wang L, Luo M-X, Yu X (2021). Programmably tiling rigidified DNA brick on gold nanoparticle as multi-functional shell for cancer-targeted delivery of siRNAs. Nat Commun.

[177] Yang Y, Han Y, Sun Q, Cheng J, Yue C, Liu Y (2021). Au-siRNA@ aptamer nanocages as a high-efficiency drug and gene delivery system for targeted lung cancer therapy. J Nanobiotechnol.

[178] Gai S, Yang G, Yang P, He F, Lin J, Jin D (2018). Recent advances in functional nanomaterials for light–triggered cancer therapy. Nano Today.

[179] Yu S, Zhou Y, Sun Y, Wu S, Xu T, Chang Y (2021). Endogenous mRNA triggered DNA-Au nanomachine for in situ imaging and targeted multimodal synergistic cancer therapy. Angew Chem Int Ed Engl.

[180] Mura S, Nicolas J, Couvreur P (2013). Stimuli-responsive nanocarriers for drug delivery. Nat Mater.

[181] Wang Y, Kohane DS (2017). External triggering and triggered targeting strategies for drug delivery. Nat Rev Mater.

[182] Kim TH, Alle M, Kim JC (2019). Oxidation-and temperature-responsive poly(Hydroxyethyl acrylate-co-phenyl vinyl sulfide) micelle as a potential anticancer drug carrier. Pharmaceutics.

[183] Wu D, Xu S, Zhang X, Li Y, Zhang W, Yan Q (2021). A near-infrared laser-triggered size-shrinkable nanosystem with in situ drug release for deep tumor penetration. ACS Appl Mater Interfaces.

[184] Xu W, Qian J, Hou G, Wang Y, Wang J, Sun T (2018). PEGylated hydrazided gold nanorods for pH-triggered chemo/photodynamic/photothermal triple therapy of breast cancer. Acta Biomater.

[185] Xu W, Wang J, Qian J, Hou G, Wang Y, Ji L (2019). NIR/pH dual-responsive polysaccharide-encapsulated gold nanorods for enhanced chemo-photothermal therapy of breast cancer. Mater Sci Eng C.

[186] Xu W, Qian J, Hou G, Wang Y, Wang J, Sun T (2019). A dual-targeted hyaluronic acid-gold nanorod platform with triple-stimuli responsiveness for photodynamic/photothermal therapy of breast cancer. Acta Biomater.

[187] Yin Z, Ji Q, Wu D, Li Z, Fan M, Zhang H (2021). H2O2-responsive gold nanoclusters @ mesoporous silica @ manganese dioxide nanozyme for “off/on” modulation and enhancement of magnetic resonance imaging and photodynamic therapy. ACS Appl Mater Interfaces.

[188] Anselmo AC, Mitragotri S (2016). Nanoparticles in the clinic. Bioeng Transl Med.

[189] El-Boubbou K (2018). Magnetic iron oxide nanoparticles as drug carriers: clinical relevance. Nanomedicine.

[190] Koonce NA, Quick CM, Hardee ME, Jamshidi-Parsian A, Dent JA, Paciotti GF (2015). Combination of gold nanoparticle-conjugated tumor necrosis factor-α and radiation therapy results in a synergistic antitumor response in murine carcinoma models. Int J Radiat Oncol Biol Phys.

[191] Libutti SK, Paciotti GF, Byrnes AA, Alexander HR, Gannon WE, Walker M (2010). Phase I and pharmacokinetic studies of CYT-6091, a novel PEGylated colloidal gold-rhTNF nanomedicine. Clin Cancer Res.

[192] Monaco H, Yokomizo S, Choi HS, Kashiwagi S (2021). Quickly evolving near-infrared photoimmunotherapy provides multifaceted approach to modern cancer treatment. VIEW.

[193] Oldenburg SJ, Boehm WN, Sauerova K, Darlington TK. Current good manufacturing practices (cGMPs) in the commercial development of nanomaterials for hyperthermia applications. Nanomater Magn Opt Hyperth Appl. Elsevier. 2019;339–53. https://linkinghub.elsevier.com/retrieve/pii/B9780128139288000132. Accessed 15 Dec 2021.

[194] Bayda S, Hadla M, Palazzolo S, Riello P, Corona G, Toffoli G (2018). Inorganic nanoparticles for cancer therapy: a transition from lab to clinic. Curr Med Chem.

[195] Stern JM, Kibanov Solomonov VV, Sazykina E, Schwartz JA, Gad SC, Goodrich GP (2016). Initial evaluation of the safety of nanoshell-directed photothermal therapy in the treatment of prostate disease. Int J Toxicol.

[196] Rastinehad AR, Anastos H, Wajswol E, Winoker JS, Sfakianos JP, Doppalapudi SK (2019). Gold nanoshell-localized photothermal ablation of prostate tumors in a clinical pilot device study. Proc Natl Acad Sci USA.

[197] Prabhakar U, Maeda H, Jain RK, Sevick-Muraca EM, Zamboni W, Farokhzad OC (2013). Challenges and key considerations of the enhanced permeability and retention effect for nanomedicine drug delivery in oncology. Cancer Res.

[198] Anselmo AC, Mitragotri S (2015). A review of clinical translation of inorganic nanoparticles. AAPS J.

[199] Khoobchandani M, Katti KK, Karikachery AR, Thipe VC, Srisrimal D, Dhurvas Mohandoss DK (2020). New approaches in breast cancer therapy through green nanotechnology and nano-ayurvedic medicine—pre-clinical and pilot human clinical investigations. Int J Nanomedicine.

[200] Cheng Y, Bao D, Chen X, Wu Y, Wei Y, Wu Z (2021). Microwave-triggered/HSP-targeted gold nano-system for triple-negative breast cancer photothermal therapy. Int J Pharm.

[201] Knights O, Freear S, McLaughlan JR (2020). Improving plasmonic photothermal therapy of lung cancer cells with anti-EGFR targeted gold nanorods. Nanomater.

[202] Poderys V, Jarockyte G, Bagdonas S, Karabanovas V, Rotomskis R (2020). Protein-stabilized gold nanoclusters for PDT: ROS and singlet oxygen generation. J Photochem Photobiol B Biol.

[203] Chuang CC, Cheng CC, Chen PY, Lo C, Chen YN, Shih MH (2019). Gold nanorod-encapsulated biodegradable polymeric matrix for combined photothermal and chemo-cancer therapy. Int J Nanomedicine.

[204] Zhang X, Xi Z, Machuki JOA, Luo J, Yang D, Li J (2019). Gold cube-in-cube based oxygen nanogenerator: a theranostic nanoplatform for modulating tumor microenvironment for precise chemo-phototherapy and multimodal imaging. ACS Nano.

[205] Cheng X, Zhou X, Xu J, Sun R, Xia H, Ding J (2021). Furin enzyme and pH synergistically triggered aggregation of gold nanoparticles for activated photoacoustic imaging and photothermal therapy of tumors. Anal Chem.

[206] Ma Y, Chen L, Li X, Hu A, Wang H, Zhou H (2021). Rationally integrating peptide-induced targeting and multimodal therapies in a dual-shell theranostic platform for orthotopic metastatic spinal tumors. Biomaterials.

[207] Yang S, Yao D, Wang Y, Yang W, Zhang B, Wang D (2018). Enzyme-triggered self-assembly of gold nanoparticles for enhanced retention effects and photothermal therapy of prostate cancer. Chem Commun.

[208] Kalinowska D, Grabowska-Jadach I, Liwinska M, Drozd M, Pietrzak M, Dybko A (2019). Studies on effectiveness of PTT on 3D tumor model under microfluidic conditions using aptamer-modified nanoshells. Biosens Bioelectron.

